# Transcriptional Network Analysis Reveals Drought Resistance Mechanisms of AP2/ERF Transgenic Rice

**DOI:** 10.3389/fpls.2017.01044

**Published:** 2017-06-15

**Authors:** Hongryul Ahn, Inuk Jung, Seon-Ju Shin, Jinwoo Park, Sungmin Rhee, Ju-Kon Kim, Woosuk Jung, Hawk-Bin Kwon, Sun Kim

**Affiliations:** ^1^Department of Computer Science and Engineering, Seoul National UniversitySeoul, South Korea; ^2^Interdisciplinary Program in Bioinformatics, Seoul National UniversitySeoul, South Korea; ^3^Department of Biomedical Sciences, Sunmoon UniversityAsan, South Korea; ^4^Graduate School of International Agricultural Technology and Crop Biotechnology Institute/GreenBio Science and Technology, Seoul National UniversitySeoul, South Korea; ^5^Department of Applied Bioscience, Konkuk UniversitySeoul, South Korea; ^6^Bioinformatics Institute, Seoul National UniversitySeoul, South Korea

**Keywords:** rice, drought stress, drought tolerance, transcription factors, network analysis, NGS data analysis

## Abstract

This study was designed to investigate at the molecular level how a transgenic version of rice “Nipponbare” obtained a drought-resistant phenotype. Using multi-omics sequencing data, we compared wild-type rice (WT) and a transgenic version (*erf71*) that had obtained a drought-resistant phenotype by overexpressing *OsERF71*, a member of the AP2/ERF transcription factor (TF) family. A comprehensive bioinformatics analysis pipeline, including TF networks and a cascade tree, was developed for the analysis of multi-omics data. The results of the analysis showed that the presence of *OsERF71* at the source of the network controlled global gene expression levels in a specific manner to make *erf71* survive longer than WT. Our analysis of the time-series transcriptome data suggests that *erf71* diverted more energy to survival-critical mechanisms related to translation, oxidative response, and DNA replication, while further suppressing energy-consuming mechanisms, such as photosynthesis. To support this hypothesis further, we measured the net photosynthesis level under physiological conditions, which confirmed the further suppression of photosynthesis in *erf71*. In summary, our work presents a comprehensive snapshot of transcriptional modification in transgenic rice and shows how this induced the plants to acquire a drought-resistant phenotype.

## Introduction

Plants respond to abiotic stress in various ways. By utilizing high-through put technologies, such as microarrays and sequencing, changes in transcript levels can be detected by measuring transcripts at multiple time points as abiotic stress continues. Thus, we now have unprecedented opportunities to associate phenotypical changes with molecular-level changes in the cell. For example, the RNA sequencing (RNA-seq) technique has been used to measure expression profiles of tens of thousands of genes (37,869 gene loci in rice with evidence of expression up until 2013; Sakai et al., 2013) simultaneously under various conditions.

One important research problem that can be investigated by using the new technologies is to understand how plants respond to drought conditions. Characterizing the effects of drought-induced stress in the agricultural business has historically been a major research topic related to the productivity of crops (Venuprasad et al., 2007; Jeong et al., 2010; Ambavaram et al., 2014). Drought has become a more serious issue due to recent dramatic climate changes. The Intergovernmental Panel on Climate Change (IPCC) estimates that the global mean temperature will increase somewhere between 1 and 4°C until 2,050, and that climate-change-related drought will significantly reduce crop productivity in Africa and Asia (IPCC, 2014). Therefore, developing strategies for handling drought stress is a very important issue in plant science and also in maintaining sustainable agricultural systems.

Leveraging technological advances, plant scientists have performed analyses of time-series gene expression profiles under drought stress and reported hundreds of drought-responsive genes in rice (Rabbani et al., 2003; Rodriguez et al., 2006; Gorantla et al., 2007; Zhou et al., 2007; Rabello et al., 2008; Degenkolbe et al., 2009). So far, plant scientists have discovered many genes that play important roles in mechanisms underlying responses to drought stress, such as stomatal closure (Huang et al., 2009), osmoprotectant synthesis (Ge et al., 2008), aquaporins (Lian et al., 2004; Guo et al., 2006), cuticular wax biosynthesis (Islam et al., 2009), and phytohormone response (Iuchi et al., 2001; Qin and Zeevaart, 2002; Du et al., 2010). Additionally, a large number of drought-induced transcription factor (TF) families are reported to control drought stress responses transcriptionally (Shinozaki and Yamaguchi-Shinozaki, 2007).

However, our knowledge of biological mechanisms related to drought stress is still limited (Debnath et al., 2011; Hadiarto and Tran, 2011). In particular, we have not investigated thoroughly how drought-stress-related genes are regulated. Understanding functions, communication, and interactions of drought-stress-related genes is critical for understanding how plants respond to drought stress. The widely used differentially expressed gene (DEG) analysis, one of the single-gene analyses, is often limited in deducing meaningful biological interpretations since it does not consider relationships among genes (Shojaie and Michailidis, 2009). Fortunately, there has been significant progress in network-based analysis techniques (Barabasi and Oltvai, 2004; Barabasi et al., 2011; Gitter et al., 2013; Ma et al., 2014) that consider complex relationships among genes. These powerful network-based analysis techniques have yet to be used for analyzing omics data for rice in the context of TFs and their target genes.

In this study, we used rice plants overexpressing *OsERF71* to study drought resistance mechanisms. The *OsERF71* gene belongs to the AP2/ERF family of transcription factors. AP2/ERF is one of the well-known TF families that regulate drought-responsive genes (Chen et al., 2002; Shinozaki et al., 2003). The members share a highly conserved DNA-binding domain known as the AP2/ERF domain (Nakano et al., 2006). Members of the AP2/ERF family have been shown to exhibit diverse functions in cellular processes, such as flower development (Elliott et al., 1996), spikelet meristem determinacy (Chuck et al., 1998), leaf epidermal cell identity (Moose and Sisco, 1996), embryo development (Boutilier et al., 2002), and stress tolerance (Dubouzet et al., 2003). Moreover, overexpression of AP2/ERF transcription factors enhances tolerance to drought in several plant species: *Arabidopsis*, tomato, wheat, and rice (Haake et al., 2002; Hsieh et al., 2002; Pellegrineschi et al., 2004; Oh et al., 2005, 2009). Recently JK Kim and his colleagues (Lee et al., 2016) reported that rice plants overexpressing the *OsERF71* TF showed enhanced survival over the wild-type under drought stress at the vegetative stage of growth and a 23–42% increase in total weight gain over the wild-type under drought stress at the reproductive stage of growth. They also investigated the target genes of *OsERF71* and found that *OsERF71* regulates *OsCINNAMOYL-COENZYME A REDUCTASE1*, a gene playing a role in lignin biosynthesis, and that rice plants overexpressing *OsERF71* formed enlarged aerenchyma and had high lignification levels.

To study drought resistance mechanisms, we investigated transcriptomic changes in response to dehydration stress between wild-type rice (WT) and *OsERF71*-overexpressing rice (*erf71*) lines. The goal was to elucidate the association between the *erf71-*specific response at the transcriptome level and the drought-resistant phenotype. For the investigation, we generated comprehensive multi-omics data, such as RNA-seq data, micro RNA (miRNA) sequencing data, and whole-genome bisulfite DNA methylation sequencing data, collected at 0, 1, and 6 h after treatment (HAT) under dehydration stress (i.e., aeration and without watering), from the two rice genotypes. A comprehensive bioinformatics analysis pipeline, including TF networks and a cascade tree, was developed and used for the analysis of the multi-omics data.

## Materials and methods

### Plant material and drought-stress treatments

We germinated seeds of WT (*Oryza sativa* ssp. *japonica* “Nipponbare”) plants and rice plants overexpressing *OsERF71* (Os06g0194000) in a “Nipponbare” background (Lee et al., 2016) on petri-dishes and then cultured them in Yoshida's solution (Yoshida et al., 1976) maintained in a temperature-controlled culture room at 29°C under 16/8-h light/dark conditions. Rice plants at the three-leaf stage were subjected to dehydration stress by removal of the culture solution. Untreated plants were used as a control. After treatment, entire plants were immediately transferred into liquid nitrogen. After a pilot experiment to measure the expression of the *Dip1* gene in WT using reverse-transcription (RT)-PCR, we selected 0, 1, and 6 HAT as time points to measure omics data. The whole plants harvested at these time points were kept frozen in liquid nitrogen until DNA/RNA extraction.

### Measuring gene expression levels

Our eight mRNA-seq data sets (0, 0.5, 1, 3, and 6 HAT mRNA-seq data for WT, and 0, 1, 6 and HAT mRNA-seq data for *erf71* upon drought stress) were processed accordingly. After total RNA was extracted using Tri-Reagent (MRC, Cincinnati, OH, USA), poly(A) RNAs were isolated using poly-T oligo-attached magnetic beads. These were next fragmented, and primer was attached to them. cDNA strands were then synthesized, and an adaptor was ligated. After PCR amplification, an mRNA library was prepared. mRNA deep sequencing (TruSeq RNA sample preparation Guide, Illumina) produced short reads. After clipping adapter/primer sequences from the short reads, we mapped them to the IRGSP1.0 reference genome (Kawahara et al., 2013; Sakai et al., 2013) using Tophat (Trapnell et al., 2009). We quantified the expression level of each gene using Cufflinks (Trapnell et al., 2010) based on gene information that was downloaded from the Rice Annotation Project website (http://rapdb.dna.affrc.go.jp/). DEG analysis was then performed using cuffdiff in the Cufflinks package.

### Network-based characterization of *OsERF71* transgenic rice

**Figure 1:**
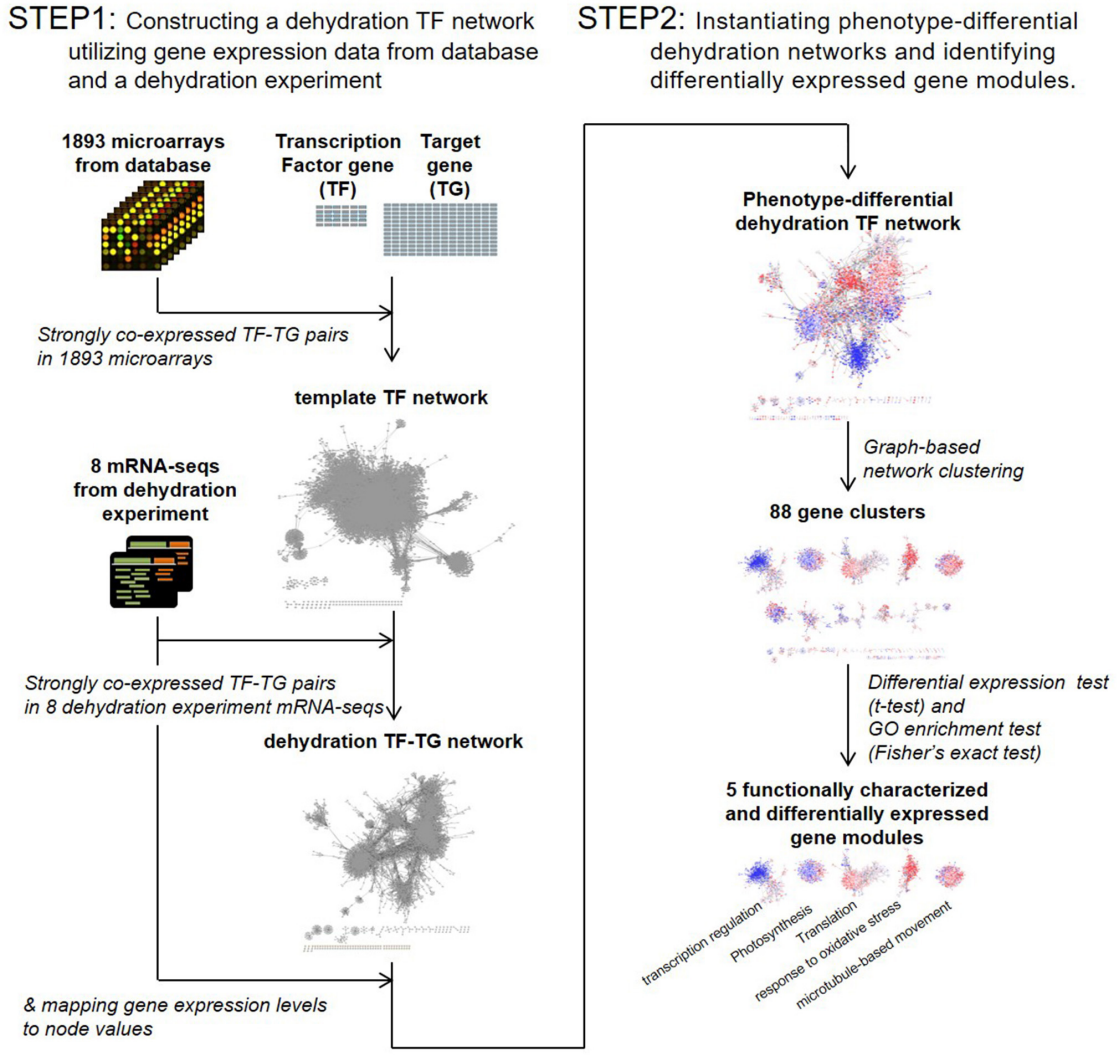
Transcription factor (TF) network analysis workflow. A template TF network was constructed by selecting strongly co-expressed TF-target gene pairs in 1,893 public domain microarrays. A dehydration TF network was then constructed by selecting strongly co-expressed TF-target gene pairs in eight dehydration experiment mRNA sequencing data sets. Phenotype-differential dehydration networks were instantiated by mapping gene expression differences to node values. Clustering analysis was then performed. Differentially expressed gene modules were selected by *t*-test, and the biological function of gene modules was characterized by gene ontology (GO) analysis.

To investigate how overexpression of the *OsERF71* TF gene affected other genes, we used a TF-network-based analysis. The challenge here was that we had limited knowledge of the relationships between the TF and its target genes. In addition, a TF activates or suppresses other TFs, producing chains of activation and suppression events. Hence, we developed a two-step bioinformatics strategy of TF-network-based analysis, as described in Figure 1.

- **Step 1**: Constructing a dehydration TF network utilizing gene expression data from databases and a dehydration experiment.
- **Step 2**: Instantiating phenotype-differential dehydration networks and identifying DEG modules.

### Network analysis step 1: constructing a dehydration TF network utilizing gene expression data from databases and a dehydration experiment

To construct a reliable dehydration TF network, we first constructed a template TF network utilizing large-scale microarray data. Before constructing a template TF network, factors, such as choice of network construction method, the data set size, and cutoff values were thoroughly investigated. A recent study investigated many network construction methods and reported that mutual-information and correlation-based methods recovered feed-forward loops most reliably (Marbach et al., 2012). Since the goal of this study was to investigate the effect of *OsERF71*-overexpression on other genes through relationships between the TF and its target genes, which can be seen as a feed-forward propagation from *OsERF71* to other genes, we selected Pearson's correlation coefficient (PCC) as the network construction method. Large-scale gene expression data sets (1,893 microarray data sets) were downloaded from the OryzaExpress Gene Expression Network website (http://bioinf.mind.meiji.ac.jp/OryzaExpress/). Probe-IDs that were used for microarray experiments were converted to gene-IDs according to a previous study (Miller et al., 2011). Since PCC is shown to converge as the sample size increases, we performed an empirical study to determine whether the data set size was beyond the convergence threshold and was sufficient to construct a template TF network robustly. Varying the number of samples, we produced different sample-size subsets of the microarray data by random sampling from the 1,893 microarray data sets. For each subset, PCCs between TF and target genes were then computed and PCC density distributions and network topologies were investigated (see “TF network construction” in Discussion). We observed that the density distributions and the network topologies converged with a sample size greater than approximately 800. This showed that 1,893 microarray data sets were sufficient to produce a robust template TF network. Recent studies that used the PCC method for biological network construction detected modular structures of genes in *Arabidopsis*, rice, and maize networks (Mao et al., 2009; Ficklin and Feltus, 2011). These studies reported that each of the modules had a specific biological function. Based on this result, we defined functionality score as follows.

$$\begin{array}{l} \text{Functionality Score}(G)=-\sum_{c_{i}\;\in C}\frac{\left|c_{i}\right|}{N}log_{10}(p_{c_{i}}) \end{array}$$

In the formula, *G* is a network and it is divided into a set of gene clusters, *C* = [*c*_1_, *c*_2_, …, *c*_*n*_], using a graph-based clustering algorithm (Blondel et al., 2008). *N* is the number of genes in the network, and *p*_*ci*_ is the *p*-value of the most significant gene ontology (GO) term in a GO enrichment test of the cluster *c*_*i*_. The functionality score measures how well a network is divided into functional gene modules. We investigated functionality score for each network constructed at different PCC cutoff values (see “TF network construction” in Discussion). The cutoff value of 0.67 was chosen because the functionality score was maximized at the cutoff value. TF-target gene pairs with strong association (|PCC| > 0.67) in the 1,893 microarray data sets were then defined as edges in the template TF network. The template TF network consisted of 10,740 genes (898 TFs and 9,842 nonTFs) and 135,550 links (4,073 TF-TF links and 131,477 TF-nonTF links). A dehydration TF network was then constructed by selecting edges in the template TF network that had strong association (|PCC| > 0.67) in eight dehydration experiment mRNA-seq data sets. The constructed dehydration TF network consisted of 7,319 genes (729 TFs and 6,590 nonTFs) and 50,672 links (1,375 TF-TF links and 49,297 TF-nonTF links). The topology of the dehydration TF network is shown in Supplemental Data Set 1. The topology of the network was visualized using Cytoscape (Saito et al., 2012).

### Network analysis step 2: instantiating phenotype-differential dehydration networks and identifying differentially expressed gene modules

In this step, our goal was to identify DEG modules between WT and *erf71* from the dehydration TF networks. To do so, we employed the following strategy.

- **Step 2–1**: Phenotype-differential dehydration TF networks were instantiated by mapping gene expression differences to node values of the dehydration TF network.
- **Step 2–2**: Graph-based network clustering broke down the phenotype-differential dehydration TF networks into several gene clusters according to connectivity.
- **Step 2–3**: Differential expression and GO enrichment tests were performed for each cluster.
- **Step 2–4**: DEG modules were selected and designated as “modules.”

In Step 2–1, we instantiated phenotype-differential dehydration TF networks by mapping gene expression differences between time points for each plant (i.e., log_2_(W1/W0) and log_2_(W6/W0)) as well as differences across plants (i.e., log_2_(E1/A0)-log_2_(E1/W0)) to nodes of the dehydration TF network, where “W” and “E” stand for WT and *erf71*, and “0”, “1,” and “6” stand for 0, 1, and 6 HAT, respectively. In Step 2–2, the phenotype-differential dehydration TF networks were broken down into several gene clusters using a multi-level network clustering method (Blondel et al., 2008) that groups highly connected nodes into a cluster of nodes. In Step 2–3, a paired sample *t*-test was performed on each cluster to determine whether each gene cluster was differentially expressed or not. Also, biological functions of each cluster were characterized by GO enrichment analysis based on Fisher's exact test. In Step 2–4, the clusters showing high-level significance (*p* < e-9) in both tests were selected and designated as “modules.”

### Measuring photosynthesis levels using a liquid CO_2_ supply system to confirm suppression of photosynthesis under drought stress

Since the suppression of photosynthesis was a key observation from analyzing sequencing data, we confirmed photosynthesis levels using a liquid CO_2_ supply system. For this confirmation experiment, we re-grew rice while measuring CO_2_ levels. Since we needed a large amount of CO_2_, we had to change experimental conditions from the three-leaf stage to the eight-leaf stage of rice and also the time points used. We selected time points corresponding to the same physiological status and degree of dehydration stress by measuring gene expression levels of *Dip1* through RT-PCR.

For photosynthesis measurements, rice plants at the eight-leaf stage were subjected to drought stress for 24, 48, and 72 h by removal of the culture solution. For measurements of Pnet and Cs, a Li-Cor 6400 photosynthesis measurement system (Li-Cor, Lincoln, NE) was used to measure gas-exchange ability. Gas-exchange measurements were performed at four stages: before treatment, and 24, 48, and 72 h after drought stress treatment. Leaves were measured in 2 × 3 cm photosynthesis measuring cuvettes under 1,500 mol m^−2^ s^−1^ light emitting diode (LED) light, 400 mmol L^−1^ CO_2_ and a cuvette temperature of 25°C. Measurements were conducted using the youngest among the 3–4 fully expanded leaves of the WT and *erf71* transgenic plants. Once a leaf was clamped in the chamber, data were automatically collected every 10 s for 3 min, while mid-point measurements were collected as representative data. A liquid CO_2_ supply system (Li-Cor 6400-01, Lincoln, NE) attached to the photosynthesis measurement system was used to measure photosynthetic changes based on changes in supplied CO_2_ concentration. CO_2_ concentration was adjusted to 400, 300, 200, 100, 50, and 0 mmol L^−1^ CO_2_. The conditions in the cuvette were 1500 mol m^−2^ s^−1^ photosynthetically active radiation, 25°C, and 30–60% relative humidity.

### Constructing a TF cascade tree with *OsERF71* as the root node from the dehydration TF networks

Dehydration differential TF networks were converted into an *OsERF7-*centric cascade tree to investigate the connection between the *OsERF71* transgene and the five modules that were identified in the TF network analysis. To achieve this goal, we added new edges of strong association (|PCC| > 0.5) to the TF network to compensate for the loss of connectivity between the *OsERF71* transgene and the five modules. The strong association value of 0.5 was chosen because it was the lower boundary that was used for network construction in previous rice species network studies (Lim et al., 2010; Zhang L. et al., 2012; Lu et al., 2013; Yang et al., 2013; Hwang et al., 2014). We confirmed that the five modules were maintained after addition of the new edges by measuring the clustering coherency value (Fowlkes and Mallows, 1983). The network was then converted into an *OsERF71*-centric cascade tree through level-wise construction of relationships starting at *OsERF71*. In this process, *OsERF71* was located to the top of the tree and the direct target genes of *OsERF71* in the TF network were then connected with *OsERF71* in the cascade tree and located at the level below in the tree. This process was performed repeatedly for all genes of the five modules. The full landscape of interactions in the constructed tree was very complex and it included multiple paths to a single gene and back edges (i.e., the edges from the TF to the target gene of previous levels). This complex relationship needed to be simplified to focus the effect of *OsERF71* overexpression. Therefore, we selected the single path with the greatest PCC score and removed the other paths as well as the back edges. The topology of the TF cascade tree is shown in Supplemental Data Set 2.

### Measuring miRNA expression levels and inferring miRNA-gene interaction

We prepared a small RNA library by attaching 3′ and 5′ adaptors to total RNA, amplifying them by PCR and performing gel purification. After deep sequencing (Illumina's TruSeq™ small RNA sample prep kit), 51-nt-length reads were generated. After the adaptor sequence was removed, miRanalyzer (Hackenberg et al., 2011) was used to quantify the expression of known rice miRNA sequences from the miRBase database (Kozomara and Griffiths-Jones, 2014). The number of reads matching known miRNAs divided by the total aligned reads was used to determine the expression level of miRNAs. Using psRNATarget, a webserver for predicting small RNA targets in plants (Dai and Zhao, 2011), candidate miRNA-target gene pairs were generated. For each of those candidates, the miRNA-target gene pairs showing a strong negative association of expression (PCC < −0.67) in our dehydration experiment were selected for the final miRNA-target gene interactions.

### Measuring DNA methylation levels and identifying differentially methylated regions

After isolating DNA, we performed whole-genome bisulfite sequencing from samples of WT and *erf71* collected at 0, 1, and 6 HAT. Adaptor sequences were removed from the raw reads, and the sequences were aligned to the rice genome using BS-MAP (Xi and Li, 2009). CpG sites with two reads or fewer aligned were not considered for further analysis. For each CpG site, we determined the methylation level as the number of methyl cytosines (i.e., cytosine transformed into thymine by bisulfite treatment) aligned to the site divided by the number of reads aligned to the site. This resulted in a normalized methylation level from 0 (hypo-methylated) to 1 (hyper-methylated).

We then considered a CpG site as a differentially methylated cytosine if the difference in maximum and minimum methylation levels among six samples was greater than 0.7, a measure for differential methylation in the CpG context in previous studies (Hsieh et al., 2009; Chodavarapu et al., 2012; Stroud et al., 2013), at the CpG site. The number of differentially methylated cytosines was 163,982 (1.4%) among 11,512,277 CpG sites with at least three reads mapped. We defined a differentially methylated region (DMR) as a genomic region containing more than eight differentially methylated cytosines in succession (*p* < 1.0e-12 by Poisson probability). The number of DMRs was 1,607 and they covered 0.14% (526,064 nt/373,245,519 nt) of the rice genome.

## Results

### Time-series experiments and multi-omics sequencing data

We carried out a pilot study to select proper time points for measuring omics data. Yi and colleagues (Yi et al., 2010) reported that *Dip1* (Os02g0669100) is induced upon dehydration stress treatment, reaching a peak at 2 HAT and remaining constant until 8 HAT, which could be used as a response marker for water deficiency. Thus, we measured the expression level of *Dip1* using RT-PCR at different time points during the dehydration treatment. The expression level of *Dip1* peaked at 3 HAT and continued to be expressed until 6 HAT. This was also confirmed by the mRNA-seq experiment (Supplemental Figure 1). Thus, three time points, 0, 1, and 6 HAT, before and after the peak expression level of *Dip1*, were selected to study how the plants responded to dehydration stress. We first performed mRNA sequencing for six samples, i.e., WT and *erf71* for 0, 1, and 6 HAT. We also performed small RNA sequencing and whole-genome bisulfite sequencing to see if small RNAs or methylation have any major regulatory roles under drought stress in addition to TFs. In summary, we generated three types of omics datasets (mRNA-seq, small RNA sequencing, and whole-genome bisulfite sequencing) from the two rice plants at three time points under dehydration stress.

### Gene expression profile comparison between WT and *OsERF71*-overexpressing rice

**Figure 2:**
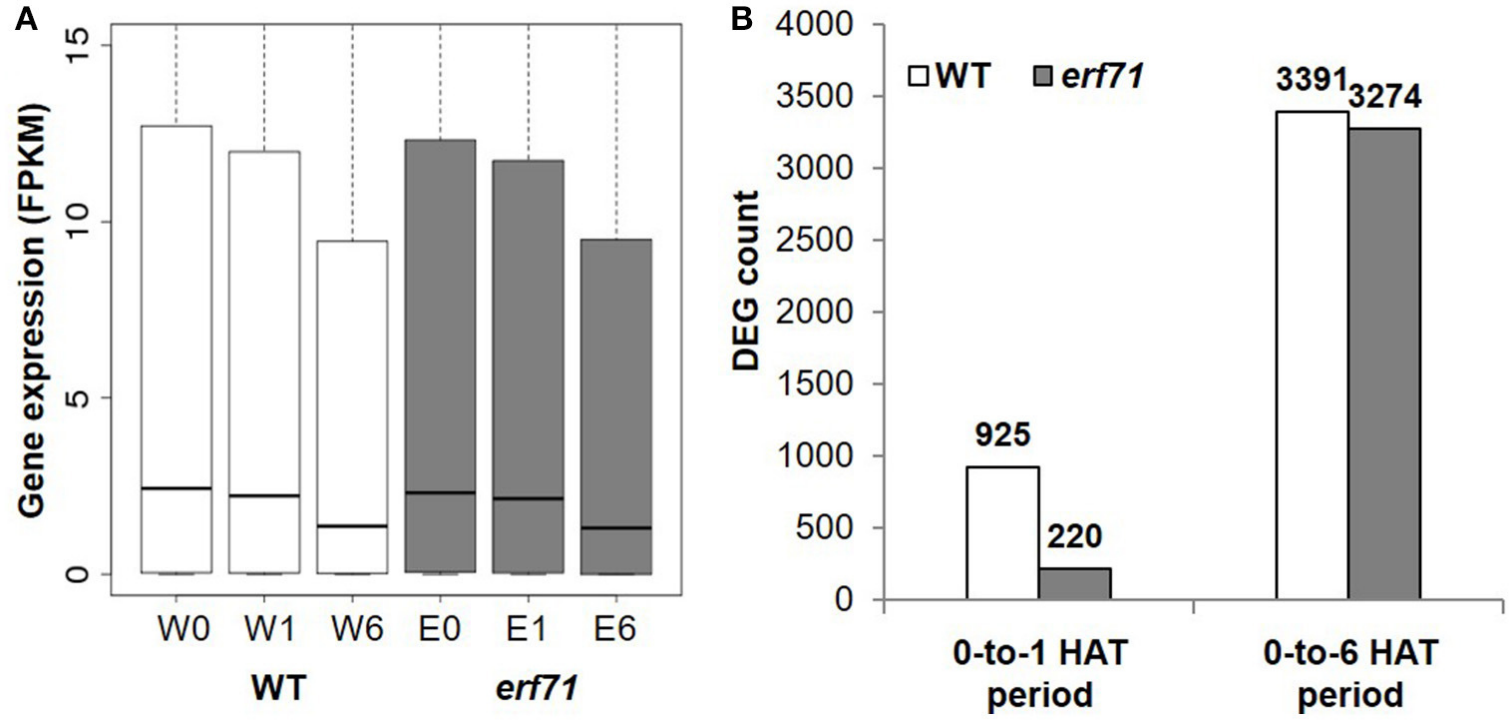
Gene expression profiles under dehydration stress. **(A)** Gene expression levels in FPKM (Fragments Per Kilobase of exon per Million fragments mapped) for six samples (i.e., two plants for three time points): WT (white) and *erf71* (gray) for 0, 1, and 6 h after treatment (HAT). Medians of gene expression (black lines in the center of the boxes) decrease as the dehydration stress continued. **(B)** The number of differentially expressed genes (DEGs) in WT (white) and *erf71* (gray). In the 0-to-1 HAT period, the number of DEGs in the WT (925 genes) is much greater than that in *erf71* (220 genes).

A total of 18,567 and 18,555 genes with FPKM (Fragments Per Kilobase of exon per Million fragments mapped) greater than 1.0 were present in WT and *erf71* samples, respectively. The overall expression level of genes decreased as the dehydration stress continued in both WT and *erf71* (Figure 2A). The *p*-values were 2e-45, 1e-434, 2e-80, and 1e-348 for the paired *t*-tests with H1: W0 > W1, W1 > W6, E0 > E1, and E1 > E6, respectively, where “W” and “E” stand for WT and *erf71*, and “0”, “1,” and “6” stand for 0, 1, and 6 HAT. This observation indicated that both WT and *erf71* responded to dehydration by lowering gene expression levels globally. DEG analysis determined DEGs as genes being differentially expressed with *p* < 0.05 in statistical tests using Cuffdiff (Trapnell et al., 2010) and showed that the number of DEGs increased more than 3-fold between the 0-to-1 HAT period and the 0-to-6 HAT period in both plants (Figure 2B). These two results showed that the transcriptomes of both plants were affected gradually as dehydration stress continued.

However, *erf71* showed a lesser degree of change in gene expression under dehydration stress in the 0-to-1 HAT period. The decrease in global gene expression in *erf71* was smaller than that in WT with a significance of *p* < 0.05 by paired *t*-test with H1: *log*(W1/W0) < *log*(E1/E0). Most prominently, the difference in the number of DEGs between WT and *erf71* was more distinctive in the early response phase (i.e., the 0-to-1 HAT period) than in the late response phase (i.e., the 0-to-6 HAT period). The number of DEGs in *erf71* (220 genes) was four times smaller than that in WT (925 genes) in the 0-to-1 HAT period. These results suggest that *erf71* was less sensitive to dehydration stress at the global transcriptome level in the early response phase (i.e., the 0-to-1 HAT period).

We next performed a GO enrichment analysis of DEGs in the 0-to-1 HAT period by employing Fisher's exact test. We identified 16 and 10 GO terms being enriched with *p* < 0.05 for WT and *erf71*, respectively. Five GO terms were commonly found in WT and *erf71*, such as “oxidation-reduction process” and “protein ubiquitination”. Eleven GO terms were specific to WT, whereas five were specific to *erf71* (Supplemental Table 1). However, the GO terms that were specifically enriched to WT or *erf71* were not intuitive for elucidating the difference of response between WT and *erf71*, which showed the limitations of the DEG-based analysis. Recent studies reported that network analysis has benefits for analyzing genomics data at the level of individual genes (i.e., DEG analysis) because it considers relationships between genes (Chi et al., 2014) and results are often easier to interpret and potential causal mechanisms can be identified (The Mutation and Pathway Analysis Working Group of the International Cancer Genome, 2015). Thus, we developed and performed a systemic bioinformatics pipeline that utilizes a transcriptional network to characterize the difference in gene expression response between WT and *erf71*. Especially, we focused on gene regulation mechanisms by TFs since our goal was to investigate the transcriptomic influence affected by the overexpression of *OsERF71*, a TF gene.

### Network-based characterization of *OsERF71*-overexpressing rice

The goal of this step was to characterize the differences in gene expression response between WT and *erf71* utilizing a TF-target gene regulatory network (TF network hereafter). In a TF network, nodes are genes (TFs and nonTFs) and edges are connections between TFs and potential target genes including TFs. Our TF network did not include edges between nonTF genes since we focused on transcription-factor-centered regulation. To achieve this goal, we developed and implemented a bioinformatics pipeline for TF network analysis (see “Network-based characterization of *OsERF71* transgenic rice” in Materials and methods).

**Figure 3:**
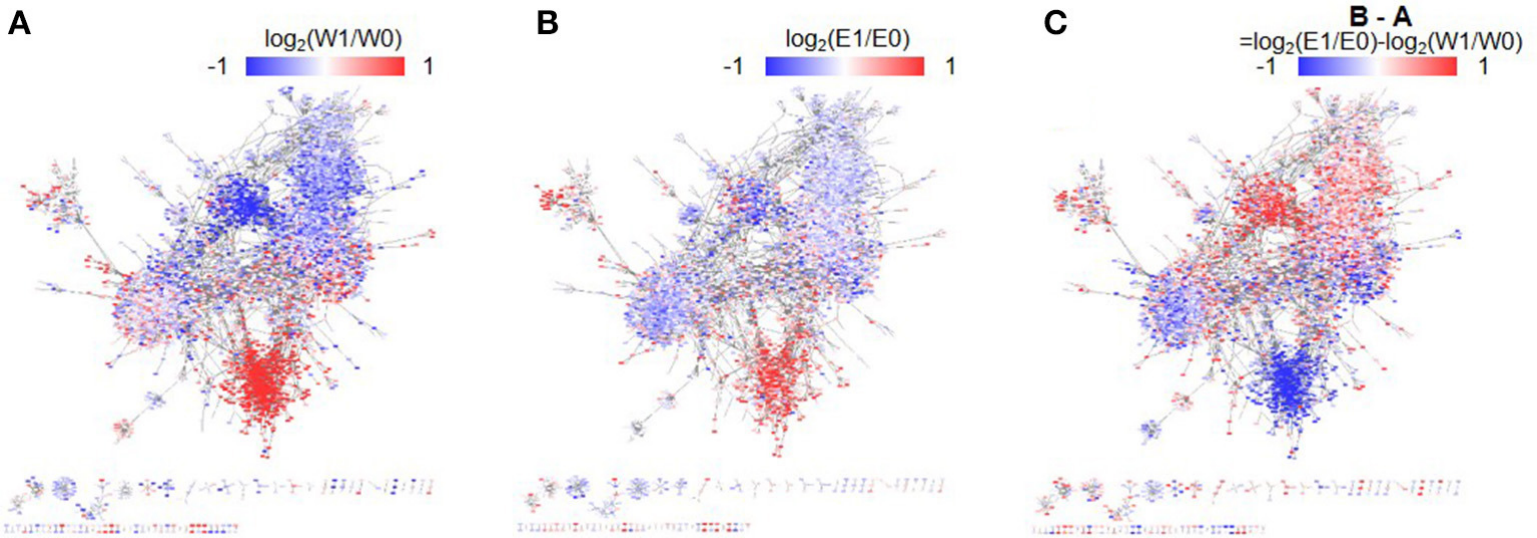
Phenotype-differential dehydration transcription factor (TF) networks. The three dehydration differential networks were instantiated by mapping gene expression differences to node values of the dehydration TF network. The node values are represented by red-white-blue-gradation. The two time-point differential networks **(A,B)** were instantiated by mapping gene expression differences between time points, such as log_2_(W1/W0) and log_2_(E1/E0), respectively, where “W” and “E” stand for WT and *erf71*, and “0”, “1”, and “6” stand for 0, 1, and 6 h after treatment. In these networks, the red/blue color represents up-/down-regulation of gene expression under dehydration stress. A phenotype-differential network **(C)** was instantiated by mapping gene expression differences between the two rice plants, such as log_2_(E1/E0)-log_2_(W1/W0). In this network, the red/blue color represents relative up-/down-regulation of gene expression in *erf71* compared with WT. The dehydration differential TF networks showed gene cluster structures distinctively, where a gene cluster indicates a sub-network with member genes highly connected to each other. They also showed a trend that member genes within gene clusters had common gene expression difference patterns.

A template TF network was constructed by selecting TF-target gene pairs with strong associations (|PCCs| > 0.67) in eight mRNA-seq data sets from our experiment and in 1,893 microarray data sets in the public domain. Differences in gene expression levels (i.e., log_2_ fold change) were then assigned to the genes in the network. Figures 3A,B shows the constructed dehydration TF networks of WT and *erf71*, respectively, where red/blue dots denote up/down-regulated genes before and after dehydration stress (i.e., 0 HAT vs. 1 HAT). Figure 3C shows a phenotype-differential TF network where red/blue dots denote relatively differentially regulated genes between WT and *erf71*. In other words, red dots indicate genes that are relatively up-regulated (more up-regulated or less down-regulated) in *erf71* under dehydration stress.

**Figure 4:**
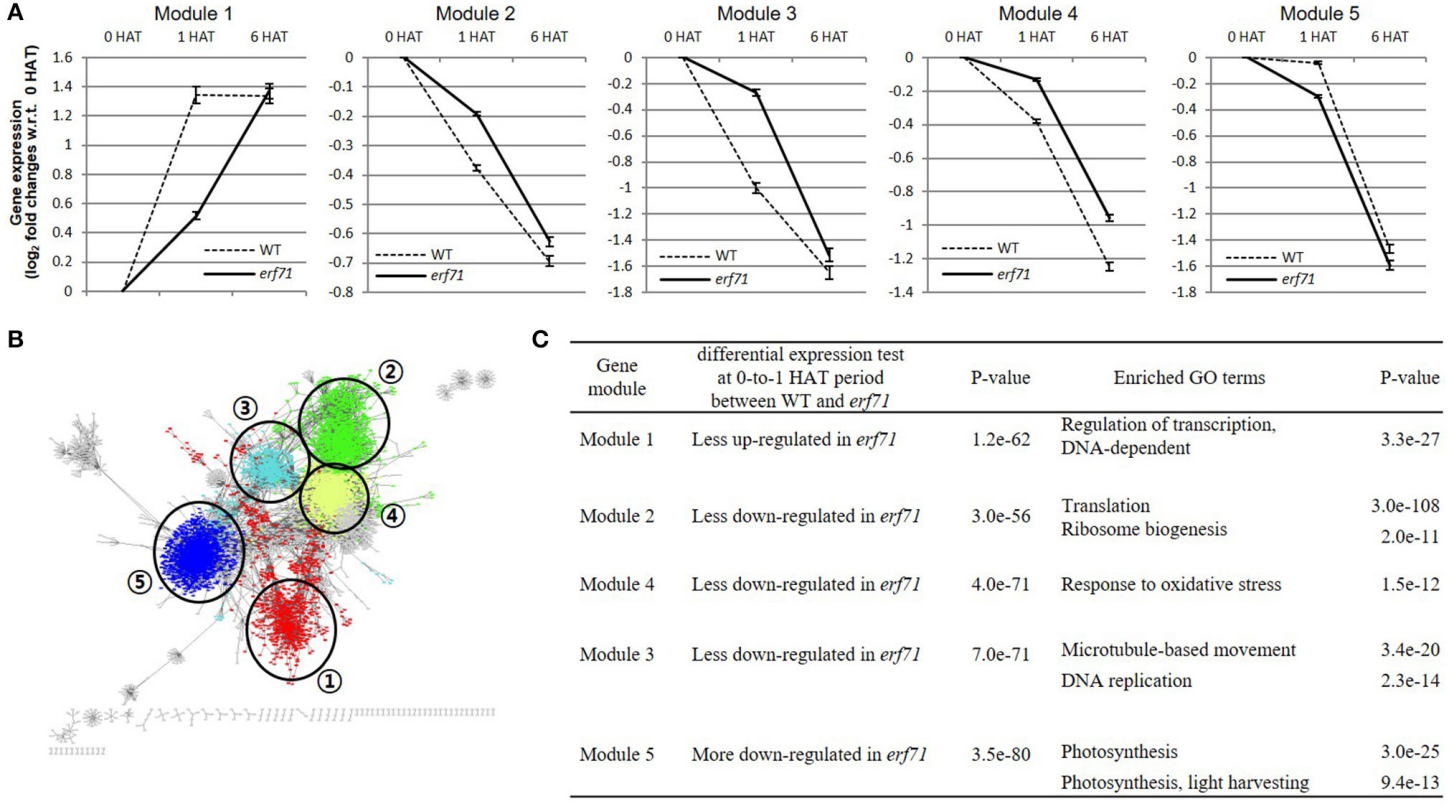
**(A)** Gene expression levels of the five differentially expressed modules. The y-axis shows the mean log_2_ fold change in gene expression level with respect to the 0 h after treatment (HAT) time point. Error bars are standard error of the means (SEMs). Module 1 was up-regulated in both types of rice but less so in *erf71*. Modules 2, 3, and 4 were down-regulated in both types of rice but less down-regulated in *erf71*. Module 5 was down-regulated in both types of rice but more down-regulated in *erf71*. **(B)** Module structures of the five modules with different colors in the dehydration network. Numbers in circles represents each module. **(C)** Results of the differential expression test and the gene ontology (GO) enrichment test of the five gene modules. The differential expression test of each module was performed using a *t*-test at 0-to-1 HAT between WT and *erf71*. The GO enrichment test was performed by Fisher's exact test. A *p*-value cutoff (*p* < 10^−9^) was used to decide differential expression and enriched GO terms.

We divided the TF network into 88 gene clusters by grouping highly connected genes into a cluster by a graph-based network clustering algorithm. We then characterized each cluster by differential gene expression and GO enrichment tests. Finally, we identified five gene clusters, with 713, 1,363, 537, 1,586, and 1,605 genes, showing high-level significance (*p* < e-9) in both tests. We designated the five clusters as “modules.” Figure 4 shows the characteristics of the five modules—plots of the expression levels, the position in the TF-TG network, and the results of GO enrichment tests.

As shown in Figure 4A, the mean expression levels of the five modules were changed in one direction (i.e., increased or decreased) as dehydration stress continued in both rice plants, but the degree of change was different between the two rice plants. Module 1 was up-regulated in both types of rice but less up-regulated in *erf71*. Modules 2, 3, and 4 were down-regulated in both types of rice but less down-regulated in *erf71*. Module 5 was down-regulated in both types of rice but more down-regulated in *erf71*.

The results of GO enrichment analysis showed that each module had different enriched GO biological process terms. Module 1 included drought-response-related TFs. A majority of genes in Module 2 were related to translation; most of the genes in Module 3 were related to response to oxidative stress; Module 4 included genes with diverse functions but many genes were related to the cell division cycle; and a large number of genes in Module 5 were related to photosynthesis. Figure 4C summarizes the results of the differential gene expression test and GO terms enrichment test for the five modules. The next section presents detailed analysis on each module.

### Analysis of module 1: drought-response-related TFs are up-regulated less in *OsERF71*-overexpressing rice

The GO term highly enriched in Module 1 was DNA-dependent regulation of transcription (GO:0006355). According to the TF list obtained from the plant TF special database, PlantTFDB (Jin et al., 2014), about a fifth of the genes in Module 1 (143/713) consisted of TFs: WRKY (27), ERF (23), NAC (17), C2H2 (10), bZIP (9), bHLH (7), MYB (6), GRAS (6), HSF (5), MYB related (5), Trihelix (4), C3H (4), HD-ZIP (2), Dof (2), NF-YB (2), SBP (2), G2-like (2), ARR-B (2), NF-YC (1), CO-like (1), RAV (1), CAMTA (1), VOZ (1), ARF (1), CPP (1), and DBB (1), where the numbers in parentheses indicate the number of genes included in the module. Among them, TF families, such as WRKY, ERF, NAC, C2H2, bZIP, bHLH, and MYB are well-known drought-stress-related TF families (Chen et al., 2012; Mizoi et al., 2012; Nakashima et al., 2012, 2014; Lindemose et al., 2013; Singh and Laxmi, 2015). Moreover, alterations in the expression of 10 TFs in Module 1, Os01g0797600 (*OsAP37*) (Oh et al., 2009), Os01g0968800 (*OsDREB1F*) (Wang et al., 2008), Os02g0654700 (*OsAP59*) (Oh et al., 2009), Os03g0741100 (*OsbHLH148*) (Seo et al., 2011), Os03g0815100 (*SNAC1*) (Hu et al., 2006), Os03g0820300 (*ZFP182*) (Zhang H. et al., 2012), Os05g0322900 (*OsWRKY45*) (Qiu and Yu, 2009), Os11g0127600 (*ONAC045*) (Zheng et al., 2009), Os11g0184900 (*OsNAC5*) (Song et al., 2011), and Os12g0583700 (*ZFP252*) (Xu et al., 2008), have already been reported to produce drought-resistant phenotypes.

We conducted RT-PCR experiments to confirm the expression levels of the TFs in Module 1 that have not yet been documented to affect drought resistance. Among them, eight TF genes, Os02g0764700 (*OsERF103*), Os03g0180900 (*TIFY11C, OsJAZ2*), Os03g0327100 (*ONAC039, OsCUC1*), Os03g0820400 (*ZFP15*), Os04g0671800 (*OsC3H32*), Os04g0676700, Os06g0670300, and Os12g0123800 (*ONAC132, ONAC300*), were shown to be up-regulated in both plants but less up-regulated in *erf71* during the 0-to-1 HAT period in response to drought stress (Supplemental Figure 2). Os03g0180900 (*TIFY11C, OsJAZ2*) is a gene of the JAZ family that contains a well-conserved domain called ZIM or TIFY (Vanholme et al., 2007). It was induced under drought stress and also by the overexpression of *OsbHLH148*, a gene that causes drought tolerance when overexpressed. Also, *OsJAZ2* exhibited a weak interaction with *OsbHLH148* and has been proposed to target activation of *OsbHLH148* (Seo et al., 2011). Os03g0327100 (*ONAC039, OsCUC1*) and Os12g0123800 (*ONAC132, ONAC300*) were reported to be responsive to drought, salt, and cold stress (Fang et al., 2008). These results show that the eight TFs were differentially regulated in WT and *erf71* during the 0-to-1 HAT period and putatively related to the drought-resistance mechanism.

Genes in Module 1 were up-regulated in both WT and *erf71*, and Module 1 was the only up-regulated module among all five modules. As overall gene expression levels decreased with continued dehydration stress, indicating the suppression of various activities, up-regulation was a relatively unexpected phenomenon. We found that Module 1 contained many up-regulated DEGs (31 up-DEGs among total 112 up-DEGs in both plants in the 0-to-1 HAT period) with a significance level of *p* < 1.0e-23 by Fisher's exact test. In summary, the expression level of TF genes was increased in our dehydration response network, and those TF genes seemed to form a modular structure in the network clustering analysis, suggesting that there might be particular biological functions of the TF module. This observation needs further investigation, which was beyond the scope of this study.

*OsERF71*, the overexpressed gene, was present in Module 1 and it had three direct neighbors (Os03g0701700, Os10g0346600, and Os11g0157200) in the module. Genes in Module 1 were directly connected to the transgene, unlike those in the other modules.

Although gene expression levels increased in Module 1, the degree of change differed between WT and *erf71*. Gene expression increased less in *erf71* in the early response phase (i.e., the 0-to-1 HAT period). This trend, relatively small gene expression changes in *erf71* in the early response phase, was observed consistently in the results of other analyses: the number of DEGs was smaller in *erf71*, and the magnitude of change in expression was smaller for genes in Modules 2, 3, and 4 and globally during the 0-to-1 HAT period.

### Analysis of modules 2, 3, and 4: critical survival-related genes are maintained

Three modules, Module 2, Module 3, and Module 4, included genes that were relatively up-regulated in *erf71* compared with WT. The enriched GO terms were genetic information processing and translation for Module 2; response to oxidative stress for Module 3; and cell cycle for Module 4. All significantly enriched GO terms were commonly related to essential biological processes for sustaining life.

In Module 3, 54 genes were related to oxidative reduction (GO:0055114) while 23 were related to response to oxidative stress (GO:0006979). Oxidation is closely related to water deficiency tolerance in plants. In particular, reactive oxygen species (ROS) are known to be overproduced in response to abiotic stress. ROS are highly reactive and toxic, causing damage to proteins, lipids, carbohydrates, and DNA when they exceed the cell's antioxidant removal capacity (Miyamoto et al., 2003; Gill and Tuteja, 2010). Since those genes in *erf71* were down-regulated to a lesser extent than in WT, it is possible that *erf71* is more capable of detoxifying the rising level of oxidation, preventing severe damage to the plant.

### Analysis of module 5: expression of genes that are related to photosynthesis is down-regulated further

Module 5 consisted of genes that were down-regulated more in *erf71* compared with WT. The significant GO terms enriched in the module were related to photosynthesis (*p* < e-13). During photosynthesis, the plant synthesizes chemical compounds using energy from light. However, such photosynthetic metabolic processes require energy use by the plant. For example, toxic elements are generated as a subsidiary product that must be detoxified, requiring the production of anti-toxic elements by the plant. Thus, maintaining such photosynthetic metabolism during a critical situation, such as dehydration, will hinder the survival of the plant (Ramachandra Reddy et al., 2004). In our analysis, *erf71* transgenic rice showed strong down-regulation of gene expression levels of photosynthetic genes compared with WT, suggesting that *erf71* was possibly able to shut down photosynthesis mechanisms in response to dehydration stress.

### Photosynthesis is suppressed physiologically further in *OsERF71*-overexpressing rice

**Figure 5:**
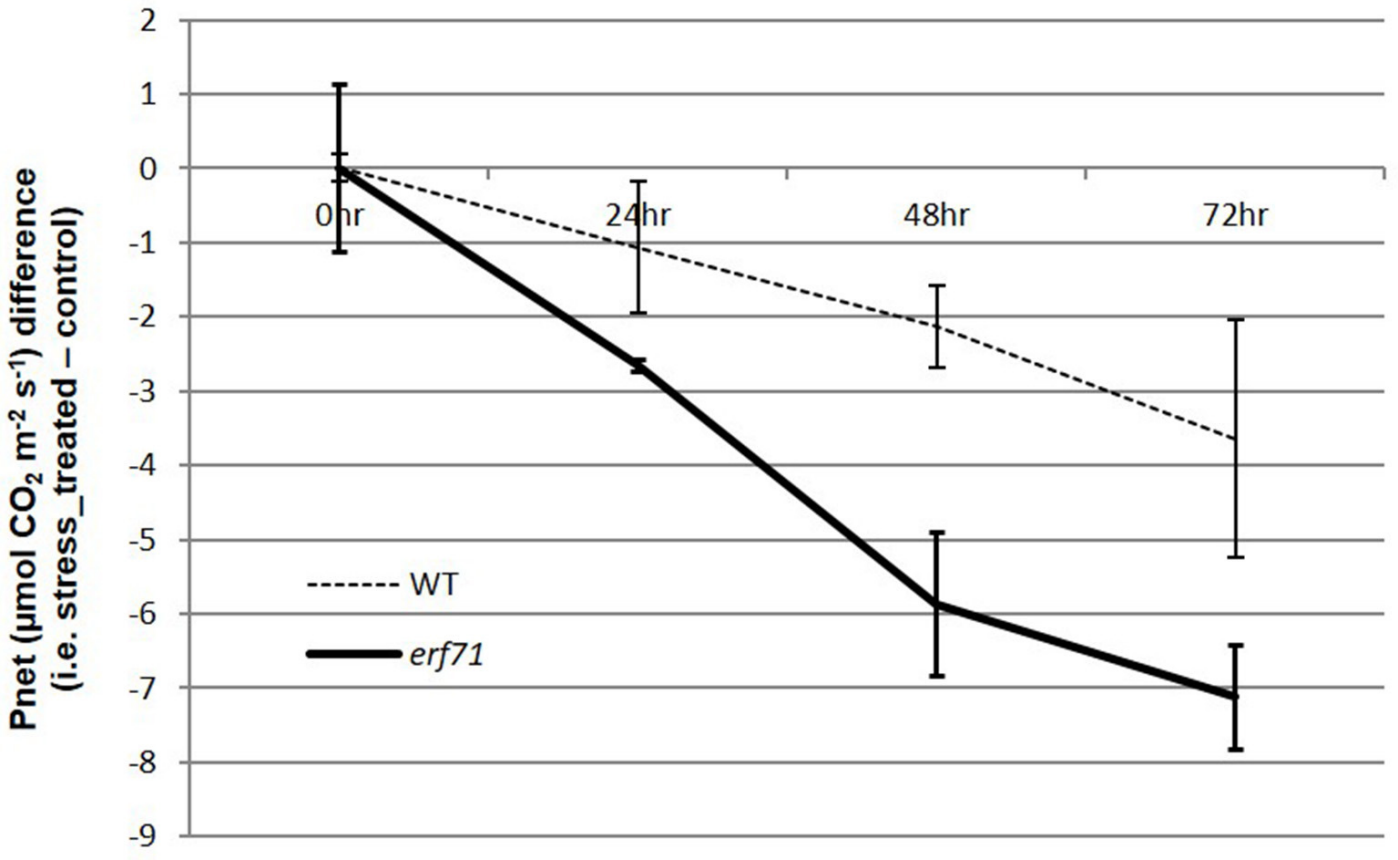
Differences in net photosynthesis levels in WT and *erf71* plants under drought stress treatment. The net photosynthesis levels were measured for WT and *erf71* at four time points under drought stress and then normalized with respect to the control sample (i.e., stress treated sample–control sample). Error bars are pooled standard error of the means (pooled SEMs). The net photosynthesis level was down-regulated in both types of rice but more so in *erf71*.

Smaller changes in gene expression in *erf71* in the early response phase (i.e., the 0-to-1 HAT period) were observed consistently. For instance, the number of DEGs was smaller in *erf71*, and the magnitude of the decrease in expression was smaller in *erf71* when considering all genes. TF network analysis also showed that genes in Modules 2, 3, and 4 were related to survival-associated biological functions under stress conditions, such as microtubule-based movement, translation, and response to oxidative stress, and these were down-regulated less in *erf71* compared with WT. This observation is intuitive since maintaining gene expression levels of survival-related genes promotes the dehydration-resistant phenotype. However, genes in Module 5 that were related to photosynthesis showed a greater response in *erf71* (i.e., the genes in Module 5 were down-regulated more in *erf71*). Since this was a key observation in this study, we measured the photosynthetic levels of WT and *erf71* plants under dehydration stress through an experiment at the physiological level. The experiment confirmed that net photosynthesis levels decreased in both plants but with greater magnitude (2-fold) in *erf71* (Figure 5).

### *OsERF71* cascade tree analysis at 1 and 6 HAT

**Figure 6:**
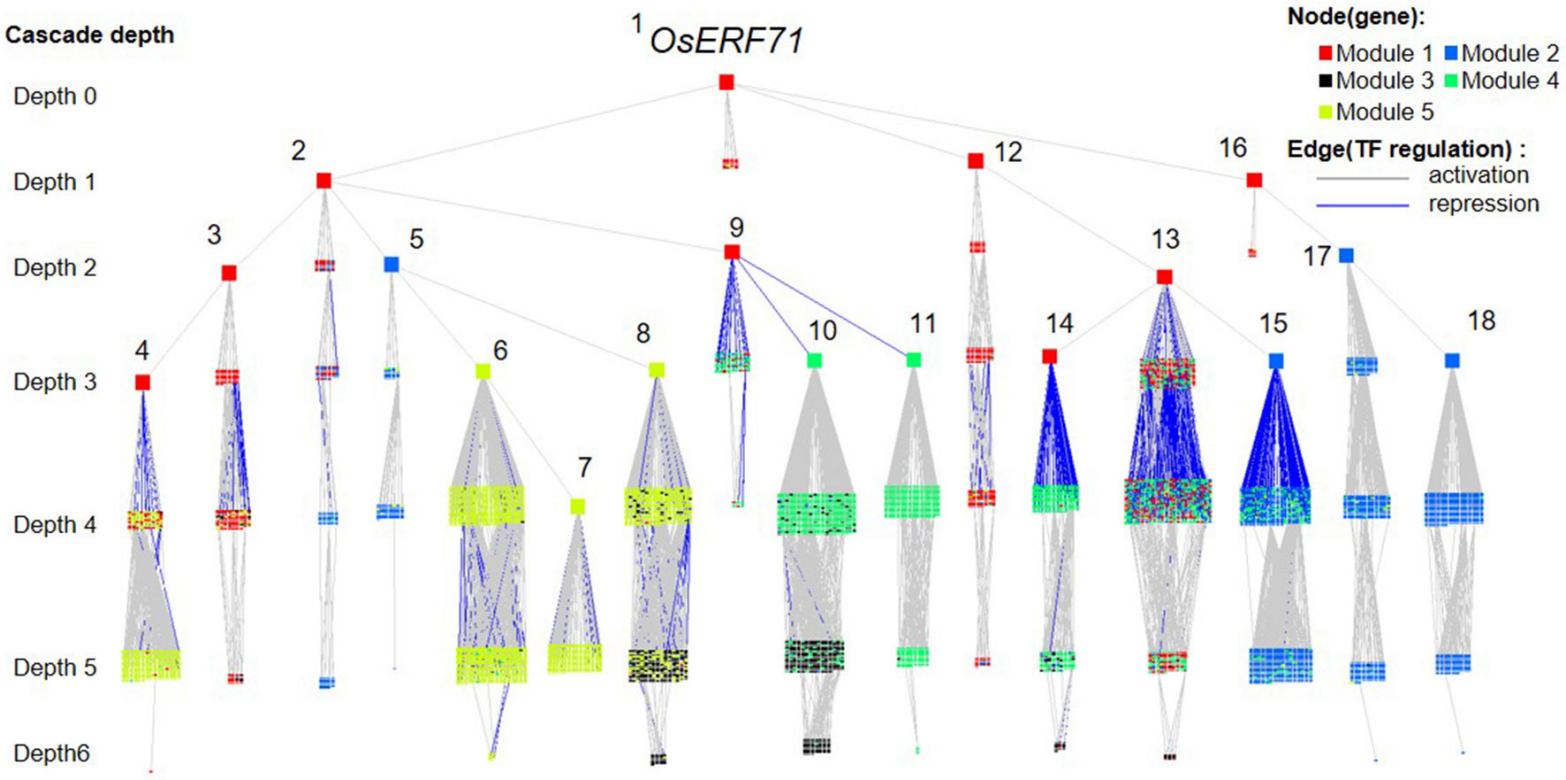
*OsERF71* cascade tree. This tree structure network was created by transforming the transcription factor (TF) regulatory network in Figure 3 into an *OsERF71*-rooted cascade tree. Nodes (*n* = 5,804) are TF or nonTF genes that are colored according to modules. Edges are TF regulatory relationships between pairs of genes that are colored according to positive/negative correlation. The cascade depth level on the left indicates the number of edges in the shortest path from *OsERF71* to that point. Hub TFs that have more than 200 genes in their downstream are highlighted by using large node sizes with gene numbers from 1 to 18, whose corresponding gene IDs are Os06g0194000, Os07g0583700, Os08g0157900, Os04g0543500, Os03g0854500, Os06g0105800, Os03g0711100, Os03g0680800, Os03g0795900, Os06g0140400, Os01g0211800, Os12g0597700, Os03g0318600, Os04g0676700, Os10g0561400, Os06g0152200, Os11g0544700, and Os07g0496300, respectively. This network shows a holistic picture of potential regulatory paths to the five gene modules.

We investigated the effects of overexpression of *OsERF71* in *erf71* by converting the differential TF regulatory network of five gene modules described in the previous section into an *OsERF71*-transgene cascade tree. The *OsERF71*-transgene cascade tree is a tree graph structure with *OsERF71* as the root node and putative downstream genes in the five modules as child nodes. The network was transformed into a tree by applying the single-source shortest TF regulatory path strategy (see “Constructing TF cascade tree with *OsERF71* as the root node from dehydration differential TF networks” in Materials and methods). The resulting *OsERF71* cascade tree is shown in Figure 6. We defined “depth” in the tree as the number of edges in the shortest path from *OsERF71* to the gene. Small-world networks are known to have a property called six degrees of seperation, in which all nodes in the network are connected to each other within a few steps. The cascade tree preserves this property since *OsERF71* is connected to all the genes within six depth levels in our cascade tree. In addition, we found in the cascade tree, major paths starting at the *OsERF71* transgene followed by regulatory hub TFs. Here, regulatory hub TFs (described by the large rectangle nodes in Figure 6) indicated transcription factors that had a large number of genes (more than 200 genes) in their downstream in the cascade tree. Moreover, we observed that there were nine dominant major paths covering the majority of genes of each module in the cascade tree. For instance, 79% of the genes in Module 5 were downstream of two major regulatory paths. One of the paths included 56% of the genes of Module 5. The upstream of the path consisted of a chain of hub TFs, Os06g0194000, Os07g0583700, Os03g0854500, and Os06g0105800, which are tagged by numbers 1, 2, 3, and 4 in Figure 6. The major regulatory paths are summarized in Table 1. We discuss regulation of the modules in detail in “Potential regulatory paths to the five functional modules” in the Discussion.

**Table 1:** The major regulatory paths to the five modules that were observed in the *OsERF71* cascade tree. Nine major regulatory paths that covered more than 10% of genes in the modules were observed in the *OsERF71* cascade tree.

**No**	**Gene number**	**Gene ID**	**Gene descriptions**	**Coverage (% of Module)**	**The most significantGO term**	***P*-value**
1	**1**	**Os06g0194000**	***OsERF71***	66 % of Module 1	Translation	2.1e-55
	**12**	**Os12g0597700**	**Similar to WRKY2**			
2	**1**	**Os06g0194000**	***OsERF71***	21 % of Module 1	Translation	1.4e-10
	**2**	**Os07g0583700**	**WRKY transcription factor 78**			
	**3**	**Os08g0157900**	**Similar to NAM protein**			
3	**1**	**Os06g0194000**	***OsERF71***	56 % of Module 5	Photosynthesis	2.6e-21
	**2**	**Os07g0583700**	**WRKY transcription factor 78**			
	5	Os03g0854500	Similar to Heat shock transcription factor 31			
	6	Os06g0105800	Homeodomain-like containing protein.			
4	**1**	**Os06g0194000**	***OsERF71***	23 % of Module 537 % of Module 3	Citrate transport	3.9e-04
	**2**	**Os07g0583700**	**WRKY transcription factor 78**			
	5	Os03g0854500	Similar to Heat shock transcription factor 31			
	8	Os03g0680800	Similar to cDNA clone: J013124I05			
5	**1**	**Os06g0194000**	***OsERF71***	46 % of Module 2	Translation	7.9e-10
	**16**	**Os06g0152200**	**Zinc-finger protein R2931**			
	17	Os11g0544700	Repressor protein.			
6	**1**	**Os06g0194000**	***OsERF71***	35 % of Module 2	Translation	2.4e-100
	**12**	**Os12g0597700**	**Similar to WRKY2**			
	**13**	**Os03g0318600**	**Similar to Transcription factor HBP-1b**			
	15	Os10g0561400	Similar to Transcription factor MYBS3.			
7	**1**	**Os06g0194000**	***OsERF71***	51 % of Module 327 % of Module 4	Response to oxidative stress	1.9e-12
	**2**	**Os07g0583700t**	**WRKY transcription factor 78**			
	**9**	**Os03g0795900**	**Similar to Heat shock transcription factor 31**			
	10	Os06g0140400	Similar to HAHB-6			
8	**1**	**Os06g0194000**	***OsERF71***	19 % of Module 4	Lipid metabolic process	2.3e-05
	**2**	**Os07g0583700**	**WRKY transcription factor 78**			
	**9**	**Os03g0795900**	**Similar to Heat shock transcription factor 31**			
	11	Os01g0211800	Similar to VirE2-interacting protein VIP1.			
9	**1**	**Os06g0194000**	***OsERF71***	14 % of Module 4	DNA replication	2.3e-06
	**12**	**Os12g0597700**	**Similar to WRKY2**			
	**13**	**Os03g0318600**	**Similar to Transcription factor HBP-1b**			
	**14**	**Os04g0676700**	**Similar to H0101F08.8 protein**			

*Gene numbers in the second column indicate the numbers given in Figure 6. The gene IDs of TFs in the paths are shown in the third column, where bold text indicates that the genes belong to Module 1. The most enriched GO term and its p-value for the downstream genes in each regulatory path are described in right-most column*.

### Multi-omics data analysis of differential network modules

The TF network analysis suggested that the five modules in the dehydration TF network were differentially expressed between WT and *erf71* in response to dehydration stress. To further investigate the regulation of the five modules in the dehydration TF network, we generated and analyzed the DNA methylome and miRNA sequencing data measured at 0, 1, and 6 HAT. DNA methylation sequencing data was used to investigate whether expression of genes in the five modules in the dehydration TF network was affected by DNA methylation, i.e., TF-DNA interaction, especially in the promoter regions. In addition, we also used miRNA sequencing data to investigate whether these genes were affected by miRNAs.

### DNA methylation analysis to investigate TF-DNA interaction

**Figure 7:**
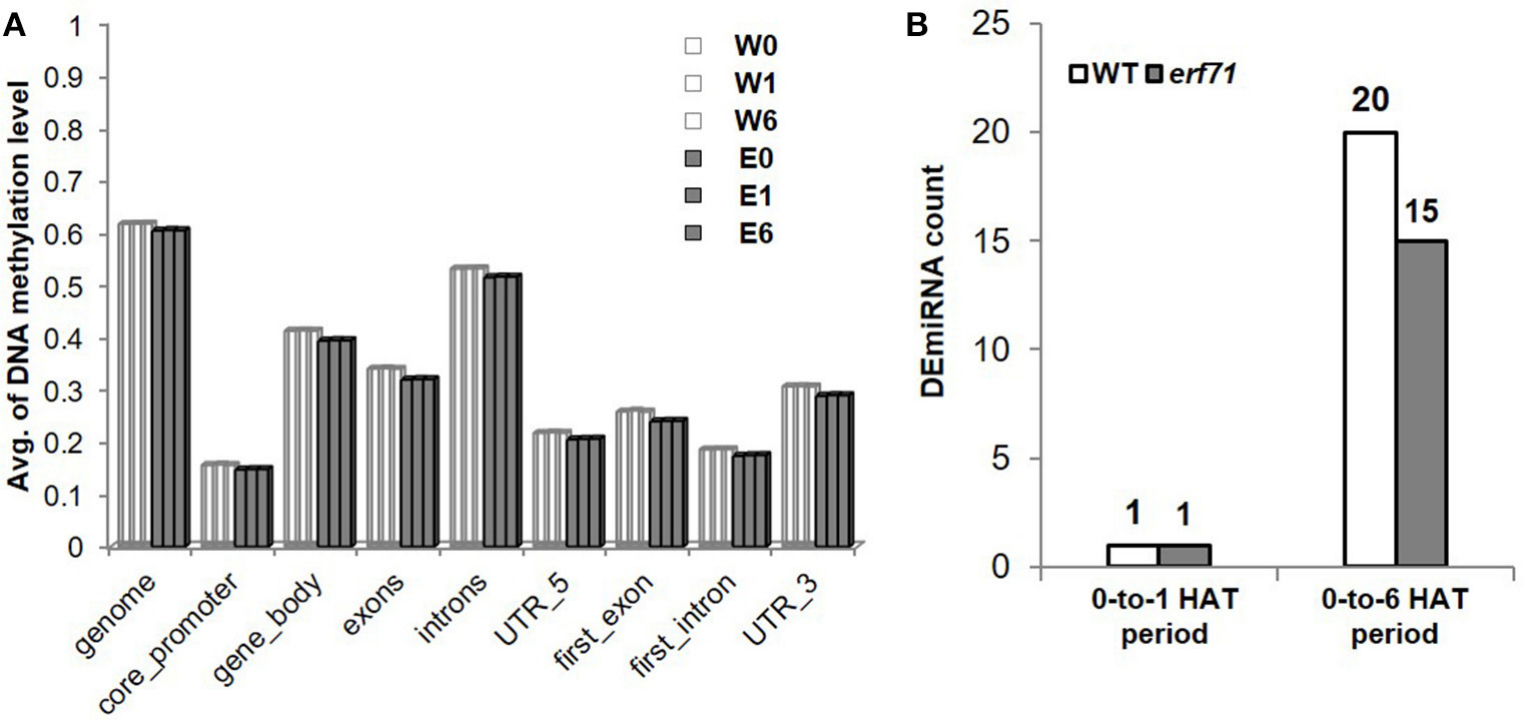
Profiles of miRNA and DNA methylation. (A) Average DNA methylation level of CpG sites in genomic regions. This shows that DNA was hypomethylated in *erf71* but not significantly changed as the dehydration stress continued. **(B)** The number of differentially expressed micro RNAs (DEmiRNAs) at 0-to-1 and 0-to-6 h after treatment periods.

Whole-genome bisulfite sequencing was performed at 0, 1, and 6 HAT in WT and *erf71* rice plants. Analysis of DNA methylation profiles showed that the average methylation levels in *erf71* were slightly lower than those in WT at the whole-genome level. Meanwhile, differences in methylation level between time points were not seen (Figure 7A), which suggested that DNA methylation did not change over the time course of dehydration stress. We then investigated differences in the methylome between WT and *erf71* and found 1,607 DMRs with *p* < e-12 under Poison' distribution (see “Identifying differentially methylated regions” in Materials and methods). The DMRs covered 0.15% of the genome, and the majority of DMRs (1,436 DMRs) were hypomethylated in *erf71*. This result is consistent with studies observing that transgenic plants show stable hypomethylation on overall DNA compared with non-transgenic plants (Stroud et al., 2013; Stelpflug et al., 2014).

Searching for overlaps between promoters of genes and DMRs, we found that promoters of 494 genes (1.3% of total genes) overlapped with DMRs. Among them, 62 genes belonged to the five modules (2, 15, 5, 9, and 21 genes to Modules 1 to 5, respectively). From the Fisher's exact test, the hypothesis that there was a greater number of DMR-overlapping genes in the five modules vs. DMR-overlapping genes not in the five modules was not significant (i.e., *p* > 0.05), as shown in Table 2. This suggested that gene expression differences of the modules were not likely due to differences in DNA methylation.

**Table 2:** Results of the association test between differential DNA methylation and the five gene modules.

**Gene module**	**DMR genes in module**	**Genes in module**	**DMR genes not in module**	**Genes not in module**	***p*-value**
Module 1	2	713	492	37,156	1.00
Module 2	15	1,363	479	36,506	0.78
Module 3	5	537	489	37,332	0.83
Module 4	19	1,586	475	36,283	0.68
Module 5	21	1,605	473	36,264	0.53

*After identifying differentially methylated regions (DMRs), genes whose promoters overlapped with DMRs were investigated. Fisher's exact test was then used to evaluate the hypothesis that DMR-overlapping genes were observed more frequently in the modules compared with not in the modules. The results showed no significant association between differential DNA methylation and the five gene modules*.

### miRNA analysis to show potential miRNA interference on modules

In addition to TFs, another major mechanism of gene control is by miRNAs. To investigate whether miRNAs affected genes in the *OsERF71* cascade tree, small RNA sequencing was performed at 0, 1, and 6 HAT in WT and *erf71* rice plants. At 1 and 6 HAT, differentially expressed miRNAs (DEmiRNAs) were identified (fold change >2 or fold change <1/2) (Figure 7B). There was one DEmiRNA during the 0-to-1 HAT period in both plants, and 20 and 15 DEmiRNAs during the 0-to-6 HAT period in WT and *erf71*, respectively. Among DEmiRNAs, we found that osa-miR166j was down-regulated during the 0-to-6 HAT period in both WT and *erf71*. Down-regulation of the MIR166 family in response to drought in rice was reported in previous studies (Zhou et al., 2010; Barrera-Figueroa et al., 2012; Cheah et al., 2015). As a next step, we performed a target gene analysis for the 32 unique DEmiRNAs and found 742 genes as potential targeted genes. Among them, 222 genes belonged to the five modules (4, 66, 10, 55, and 87 genes to Modules 1 to 5, respectively). The result of Fisher's exact test showed that DEmiRNA target genes were significantly represented in the modules (five modules and Modules 2, 4, and 5) compared with target genes not in the modules, as shown in Table 3. This suggested that gene expression differences in the modules were possibly due to regulation by miRNAs as well as TFs.

**Table 3:** Results of the association test between target genes of differentially expressed miRNAs (DEmiRNAs) and the five gene modules.

**Gene module**	**DEmiRNA genes in module**	**Genes in module**	**DEmiRNA genes not in module**	**Genes not in module**	***p*-value**
Module 1	4	713	738	37,156	1.00
Module 2	66	1,363	676	36,506	**2.0e-11**
Module 3	10	537	732	37,332	0.61
Module 4	55	1,586	687	36,283	**3.7e-05**
Module 5	87	1,605	655	36,264	**1.2e-17**

*After determining target genes of the DEmiRNAs, Fisher's exact test was used to evaluate the hypothesis that DEmiRNA target genes were observed more frequently in the modules compared with not in the modules. The results showed that the associations between DEmiRNA target genes and Modules 2, 3, and 5 were significant (Bold value indicates p < 0.05)*.

## Discussion

### TF network construction

The TF network is the major computational resource for investigating biological mechanisms under dehydration conditions. Thus, we investigated all issues related to the TF network construction thoroughly.

- Choice of the correlation-based TF network construction method.
- Effect of amount of omics data for network construction.
- Effect of cutoff values for a minimum correlation between genes.
- Comparison with other network construction and analysis methods.

### Choice of the correlation-based TF network construction method

Gene regulation network construction methods centered around TFs have been extensively studied over the years, and network-based analysis of omics data has been successful in reconstructing gene regulatory paths under specific conditions. For example, Califano and colleagues (Basso et al., 2005) demonstrated a reverse-engineered construction of regulatory networks in human B-cells using gene expression data. A recent comprehensive study (Marbach et al., 2012) of biological network construction methods classified network construction methods into several groups based on the techniques used: regression (Haury et al., 2012), mutual information (Faith et al., 2007), correlation (Butte and Kohane, 2000), Bayesian networks (Statnikov and Aliferis, 2010), other approaches (Huynh-Thu et al., 2010), and meta predictors (Greenfield et al., 2010). According to the study (Marbach et al., 2012), correlation-based network construction is effective for investigating feed-forward networks. Our study on the role of *OsERF71* in the acquisition of the drought-resistant phenotype can be seen as investigating feed-forward networks originating from *OsERF71*. Therefore, we used a method based on Pearson's correlation for network construction.

### Effect of amount of omics data for network construction

In addition to the methods used for network construction, the amount of omics data used for network construction has a significant impact on the network topology. Hence, it was important to investigate whether the omics data we used for this study was sufficient to produce a network topology invariant of the size of data set used. To investigate this, we performed comprehensive network construction experiments with varying numbers of microarray data sets, from four to 1,893, doubling the microarray data size at each round. To reduce sampling bias, we used 10 randomly sampled microarray data sets for each network experiment with *n* samples from *n* = 4 to *n* = 1,893; i.e., the network was constructed 10 times given a sample size *n*. We then found that as the sample size increased, the Pearson's correlation coefficients converged into a normal-distribution-like shape (Supplemental Figure 3). As the sample size increased, the topology differences among the 10 networks with different samples disappeared, converging into a single network topology. The network topology was the same in more than 90% of network construction experiments from sample size 800 to 1,893 (Supplemental Figure 4).

### Effect of cutoff values for a minimum correlation between genes

The network topology is determined by a cutoff value for a minimum correlation value between two genes. To choose a reliable cutoff value, we measured the functionality scores as defined in Materials and methods using varying cutoff values and then found that the cutoff value of 0.67 maximized the functionality score (Supplemental Figure 5). To investigate the effect of cutoff value, we generated networks using varying cutoff values. We observed that the five modules were consistently produced at PCC cutoff values ranging from 0.5 to 0.85 (Supplemental Figure 6). This result shows that the TF network module analysis generated robust results for varying cutoff values.

### Comparison with other network construction and analysis methods

Our choice of the correlation-based network construction method was based on a recent study by Marbach et al. (2012) that reported that correlation-based network construction is the most effective method for recovering feed-forward loops, which can be seen as a regulation mechanism of the feed-forward propagation from *OsERF71*, which was the subject of this study. However, we also examined two major network construction and analysis methods as described below.

We constructed another template TF network using a state-of-the-art gene-expression-based TF network construction method, Narromi (Zhang et al., 2013). Using 1,893 microarray data sets, Narromi produced 6,213,143 TF-target gene edges (*p* < 0.05). Edges of strong association (|PCC| > 0.67) in eight mRNA-seq data sets were then selected. The Narromi-based dehydration TF network consisted of 23,236 genes and 1,286,285 edges. Of these, 7,314 genes, and 28,616 edges were common with our correlation-based network. In the network, seven gene cluster structures were observed (Supplemental Figure 7A). Among the clusters, we found that five gene clusters were differentially expressed (Supplemental Table 2). In summary, the discovery of five functional modules was confirmed by the network constructed using Narromi.

Another TF network analysis was performed using RiceNet (Lee et al., 2011), a well-curated gene-gene interaction network. RiceNet consists of 14,949 TF-target gene edges. Since our goal was to characterize the role of a knockout TF, we excluded edges between nonTFs. Edges with strong association (|PCC| > 0.67) in eight mRNA-seq data sets were then selected. The RiceNet-induced dehydration TF network consisted of 2,345 genes and 3,667 edges. Of these, 1,234 genes and 456 edges were common with our correlation-based network. In the network, 137 gene cluster structures were observed (Supplemental Figure 7B). Among the clusters, we found that one gene cluster was differentially expressed (Supplemental Table 2). Only one network module was detected in the RiceNet network. We conjecture that this is because we excluded edges between two nonTF genes, which could affect the formation of gene clusters. In addition, RiceNet is a general template network that is not designed for condition-specific gene expression data.

### Characteristic physiological mechanisms in modules

Many genes in Module 5 were related to photosynthesis and energy-generating mechanisms: functions of genes were related to light (e.g., Os01g0764500, similar to uvrB/uvrC motif-containing protein) and photosynthesis (e.g., Os01g0773700, similar to photosystem II reaction center W protein; Os03g0267300, similar to fructose-1,6-bisphosphatase; Os12g0291100, Rubisco small subunit), energy transfer (e.g., Os03g0278900, ATPase; Os11g0661300, similar to ADP/ATP translocase-like protein), and subsequent anabolic pathways, such as those for carbohydrate (e.g., Os01g0686200, UDP-glucuronosyl/UDP-glucosyltransferase family protein), carbohydrate movement and translocation (e.g., Os03g0363500, similar to vacuolar monosaccharide symporter 1), amino acid synthesis (nitrogen fixation) (e.g., Os06g0694500, similar to nitrogen fixation like protein; Os07g0658400, similar to ferredoxin-dependent glutamate synthase), and lipid synthesis (e.g., Os02g0589000, lecithin:cholesterol acyltransferase family protein). In summary, based on functions of the genes in Module 5, biological mechanisms of Module 5 were related to energy generation, storage and transfer. Recall that genes in Module 5 were more down-regulated in *erf71* than in WT at 1 HAT. This suggests a fast and flexible response in the early stages of drought stress that may be directly and/or indirectly related to the successive physiological mechanisms observed in Modules 2, 3, and 4.

Discontinuation of water supply to the plant is known to result in loss of tension and decrease in water potential in the cells of leaves, which directly affects most metabolic pathways, particularly photosynthesis as shown above. The widely known water split process that activates an electron and releases it from the water molecules in the reaction center of PSII is affected by water shortage in photosynthetic cells. Most of the genes that code for proteins of PSII were down-regulated in both WT and *erf71*, which is consistent with our current knowledge.

ROS, including hydrogen peroxide (H_2_O_2_), superoxide anion (${\text{O}}_{2}^{-}$), hydroxyl radical (^.^OH) and singlet oxygen (^1^O_2_), are produced in chloroplasts, mitochondria, peroxisomes, cell membranes and cell wall spaces (Moradi and Ismail, 2007). Many enzymes and physical processes are involved in ROS production and scavenging. A large portion of ROS is produced by NADPH oxidase (NOX) during photosynthesis and photorespiration, and representative ROS scavenging enzymes are superoxide dismutase (SOD), catalase (CAT), peroxidase (Perox), and ascorbate peroxidase (APX) while non-enzymatic scavenging systems include various flavonoids, alkaloids, phenolic compounds, tocopherols, carotenoids, and metallothioneins (MTs) (Ueda et al., 2013; Han et al., 2014). It has traditionally been believed that under conditions of limited water supply the response of rice plants is to overcome physical and chemical damage and that the generation of ROS is the result of accumulation from cellular breakdown. However, recent studies have revealed that ROS may confer protection against water stress (Kar, 2011). ROS may be involved in homeostasis and various metabolic changes, morphological and anatomical changes, and metabolic adaptation by rice plants under water stress closely related to drought avoidance or postponement and alleviation of stress-induced cellular injuries.

### Potential regulatory paths to the five functional modules

TF network analysis showed that Module 1 was associated with drought-stress-related TFs. That is, 20% of genes in Module 1 consisted of TFs, most of which belonged to water-stress-related TF families. In addition, only Module 1 was up-regulated in both WT and *erf71* in response to dehydration stress whereas the other modules were down-regulated. Overexpressing *OsERF71* influenced Module 1 by reducing the up-regulation of expression of the member genes under dehydration stress. Analysis of an *OsERF71* cascade tree showed that TFs in Module 1 were present in every upstream of nine major regulatory paths as highlighted in bold type in Table 1. These results suggest that the down-regulation of overall gene expression is initiated upon up-regulation of the stress-induced TF module. We also suggest nine major regulatory paths determining how the downstream genes of the modules are regulated during dehydration stress treatment. However, since these were established by inference, they should be validated by further experiments. In addition, we found that miRNA was also involved in the control of gene expression of the five differentially expressed modules. For instance, we found that osa-miR319a was up-regulated under dehydration stress in *erf71*, which is consistent with the observations of a previous study (Zhou et al., 2010). The 8, 12, 2, and 5 predicted target genes of osa-miR319a were distributed in Modules 2 to 5, respectively. Regulation by both TF and miRNA as we suggest is statistically supported, but there needs to be experimental validation for each path and thus future study is required.

## Accession numbers

Raw sequencing data are available via accession ID: GSE74465 in the Gene Expression Omnibus (GEO) database.

## Author contributions

JK provided *OsERF71* transgenic rice seeds. SK and HK designed the project. HK and SS designed and conducted the drought experiments. HA and IJ processed the omics data and performed bioinformatics analysis. HA and JP performed network analysis, and SR performed miRNA analysis. WJ interpreted the network analysis results biologically. HA, IJ, SK, and WJ wrote the paper.

### Conflict of interest statement

The authors declare that the research was conducted in the absence of any commercial or financial relationships that could be construed as a potential conflict of interest.
