# Supplementary material for: Transcriptional Network Analysis Reveals Drought Resistance Mechanisms of AP2/ERF Transgenic Rice
Source: Front Plant Sci. 2017 Jun 15;8:1044. doi: 10.3389/fpls.2017.01044 (PMC5471331; doi:10.3389/fpls.2017.01044)
Supplement: Supplementary file 1 [file Table1.DOCX]

**Supplemental Table 1. Results of gene ontology enrichment analysis of differentially expressed genes of WT and *erf71* at the 0-to-1 h after treatment period.**

| Plant | GO biological process terms | #gene | P-value |
| --- | --- | --- | --- |
| WT | **GO:0055114+oxidation-reduction process** | 72 | 7.3e-05 |
|  | **GO:0006525+arginine metabolic process** | 2 | 0.001 |
|  | GO:0006355+regulation of transcription, DNA-dependent | 57 | 0.001 |
|  | **GO:0006559+L-phenylalanine catabolic process** | 3 | 0.003 |
|  | **GO:0009611+response to wounding** | 3 | 0.004 |
|  | GO:0019752+carboxylic acid metabolic process | 4 | 0.005 |
|  | GO:0005992+trehalose biosynthetic process | 4 | 0.012 |
|  | **GO:0016567+protein ubiquitination** | 8 | 0.012 |
|  | GO:0006470+protein dephosphorylation | 7 | 0.012 |
|  | GO:0015746+citrate transport | 2 | 0.017 |
|  | GO:0006541+glutamine metabolic process | 2 | 0.017 |
|  | GO:0009165+nucleotide biosynthetic process | 2 | 0.023 |
|  | GO:0006833+water transport | 3 | 0.023 |
|  | GO:0006869+lipid transport | 8 | 0.025 |
|  | GO:0006562+proline catabolic process | 1 | 0.035 |
|  | GO:0005991+trehalose metabolic process | 1 | 0.035 |
| *erf71* | **GO:0009611+response to wounding** | 4 | 1.2e-06 |
|  | GO:0006568+tryptophan metabolic process | 3 | 5.6e-05 |
|  | GO:0015977+carbon fixation | 3 | 1.1e-04 |
|  | GO:0008152+metabolic process | 20 | 0.001 |
|  | GO:0009415+response to water | 2 | 0.001 |
|  | **GO:0006559+L-phenylalanine catabolic process** | 2 | 0.003 |
|  | **GO:0055114+oxidation-reduction process** | 22 | 0.003 |
|  | **GO:0016567+protein ubiquitination** | 4 | 0.008 |
|  | **GO:0006527+arginine catabolic process** | 1 | 0.027 |
|  | GO:0006979+response to oxidative stress | 4 | 0.049 |
