# Supplementary material for: Transcriptional Network Analysis Reveals Drought Resistance Mechanisms of AP2/ERF Transgenic Rice
Source: Front Plant Sci. 2017 Jun 15;8:1044. doi: 10.3389/fpls.2017.01044 (PMC5471331; doi:10.3389/fpls.2017.01044)
Supplement: Supplementary file 2 [file Table2.DOCX]

Supplemental Table 2. Differentially expressed modules of RiceNet- and Narromi-based transcription factor networks. In previous step, five differentially expressed modules were identified on PCC-based dehydration TF network as shown in Table 2 of the main body of the text. One and five differentially expressed modules were identified (p < e-9) on RiceNet and Narromi-based dehydration TF network respectively. The bold texts indicate the enriched GO terms that were commonly found in the five differentially expressed modules of PCC-based dehydration TF network. Especially, the five modules of the Narromi-based network corresponded to the five modules of the PCC-based network in terms of both of differential expression pattern and enriched GO terms.

| modules | Differential expression t-test (at 0-to-1 HAT period compared to WT) | p-value | enriched GO terms (Top 3) | p-value |
| --- | --- | --- | --- | --- |
| RiceNet- based network module | less up-regulated in *erf71* | 5.91e-23 | **regulation of transcription, DNA-dependent** | 4.8e-08 |
|  |  |  | oligopeptide transport | 6.1e-03 |
|  |  |  | nucleotide-sugar transport | 8.6e-03 |
| Narromi- based network modules | Less up-regulated in *erf71* | 1.88e-76 | protein ubiquitination | 7.5e-05 |
|  |  |  | intracellular protein transport | 4.7e-04 |
|  |  |  | **regulation of transcription, DNA-dependent** | 5.3e-04 |
|  | Less down-regulated in *erf71* | 1.28e-53 | **translation** | 2.9e-19 |
|  |  |  | RNA processing | 3.5e-09 |
|  |  |  | DNA repair | 9.0e-09 |
|  | Less down-regulated in *erf71* | 6.3e-160 | defense response | 2.1e-09 |
|  |  |  | oxidation-reduction process | 4.8e-07 |
|  |  |  | **response to oxidative stress** | 3.6e-05 |
|  | Less down-regulated in *erf71* | 5.3e-145 | **microtubule-based movement** | 6.2e-17 |
|  |  |  | **DNA replication** | 1.6e-09 |
|  |  |  | protein polymerization | 2.7e-08 |
|  | More down-regulated in *erf71* | 2.9e-41 | **photosynthesis** | 7.8e-15 |
|  |  |  | **photosynthesis, light harvesting** | 3.4e-10 |
|  |  |  | carotenoid biosynthetic process | 1.4e-05 |
