# Supplementary material for: Transcriptional Network Analysis Reveals Drought Resistance Mechanisms of AP2/ERF Transgenic Rice
Source: Front Plant Sci. 2017 Jun 15;8:1044. doi: 10.3389/fpls.2017.01044 (PMC5471331; doi:10.3389/fpls.2017.01044)
Supplement: Supplementary file 5 [file Image1.PDF]

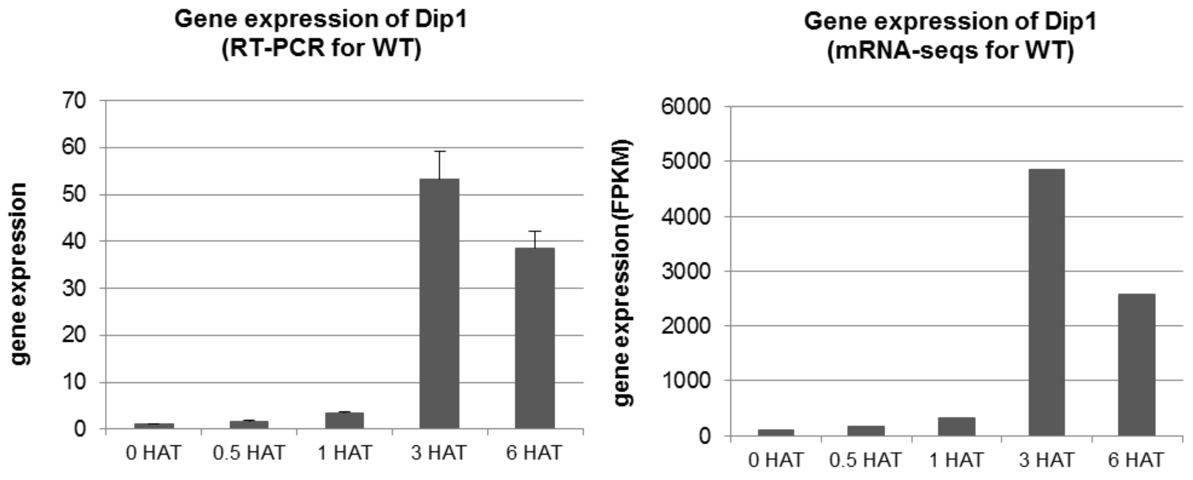

Supplemental Fig. 1. Gene expression levels of the *Dip1* gene measured by reverse-transcription (RT)-PCR and mRNA sequencing under dehydration stress. Error bars are standard error of the means (SEMs). The expression level of *Dip1* (Os02g0669100) peaked at 3 hours after treatment (HAT) under dehydration stress.
