# Supplementary material for: Transcriptional Network Analysis Reveals Drought Resistance Mechanisms of AP2/ERF Transgenic Rice
Source: Front Plant Sci. 2017 Jun 15;8:1044. doi: 10.3389/fpls.2017.01044 (PMC5471331; doi:10.3389/fpls.2017.01044)
Supplement: Supplementary file 6 [file Image2.PDF]

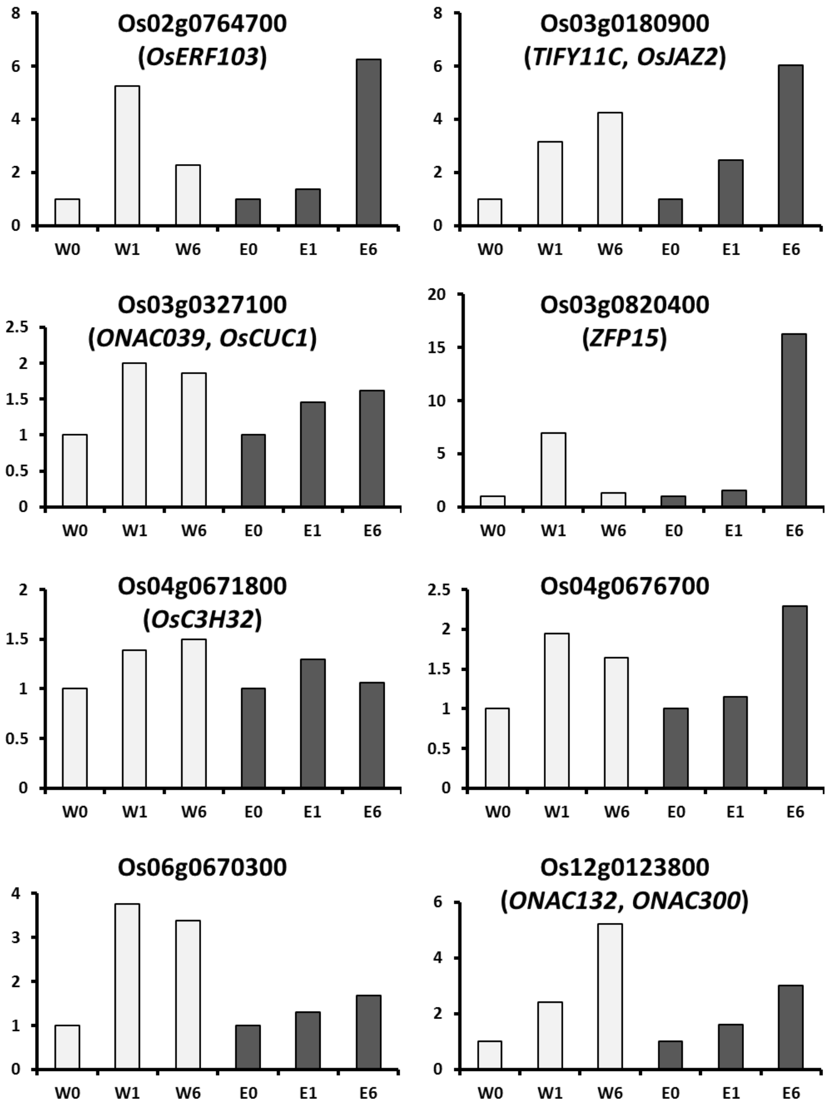

Supplemental Fig. 2. Reverse-transcription (RT)-PCR analysis of eight transcription factor genes in Module 1. We conducted RT-PCR experiment to measure the expression levels again for the TFs in Module 1 that have not been documented yet whether the transformations affect drought resistance. Among them, eight TF genes such as Os02g0764700 (*OsERF103*), Os03g0180900 (*TIFY11C*, *OsJAZ2*), Os03g0327100 (*ONAC039*, *OsCUC1*), Os03g0820400 (*ZFP15*), Os04g0671800 (*OsC3H32*), Os04g0676700, Os06g0670300 and Os12g0123800 (*ONAC132*, *ONAC300*) were shown to be up-regulated in both plants but less up-regulated in erf71 in 0-to-1 HAT period in response to drought stress as in the mRNA-seq experiment.
