# Supplementary material for: Transcriptional Network Analysis Reveals Drought Resistance Mechanisms of AP2/ERF Transgenic Rice
Source: Front Plant Sci. 2017 Jun 15;8:1044. doi: 10.3389/fpls.2017.01044 (PMC5471331; doi:10.3389/fpls.2017.01044)
Supplement: Supplementary file 7 [file Image3.PDF]

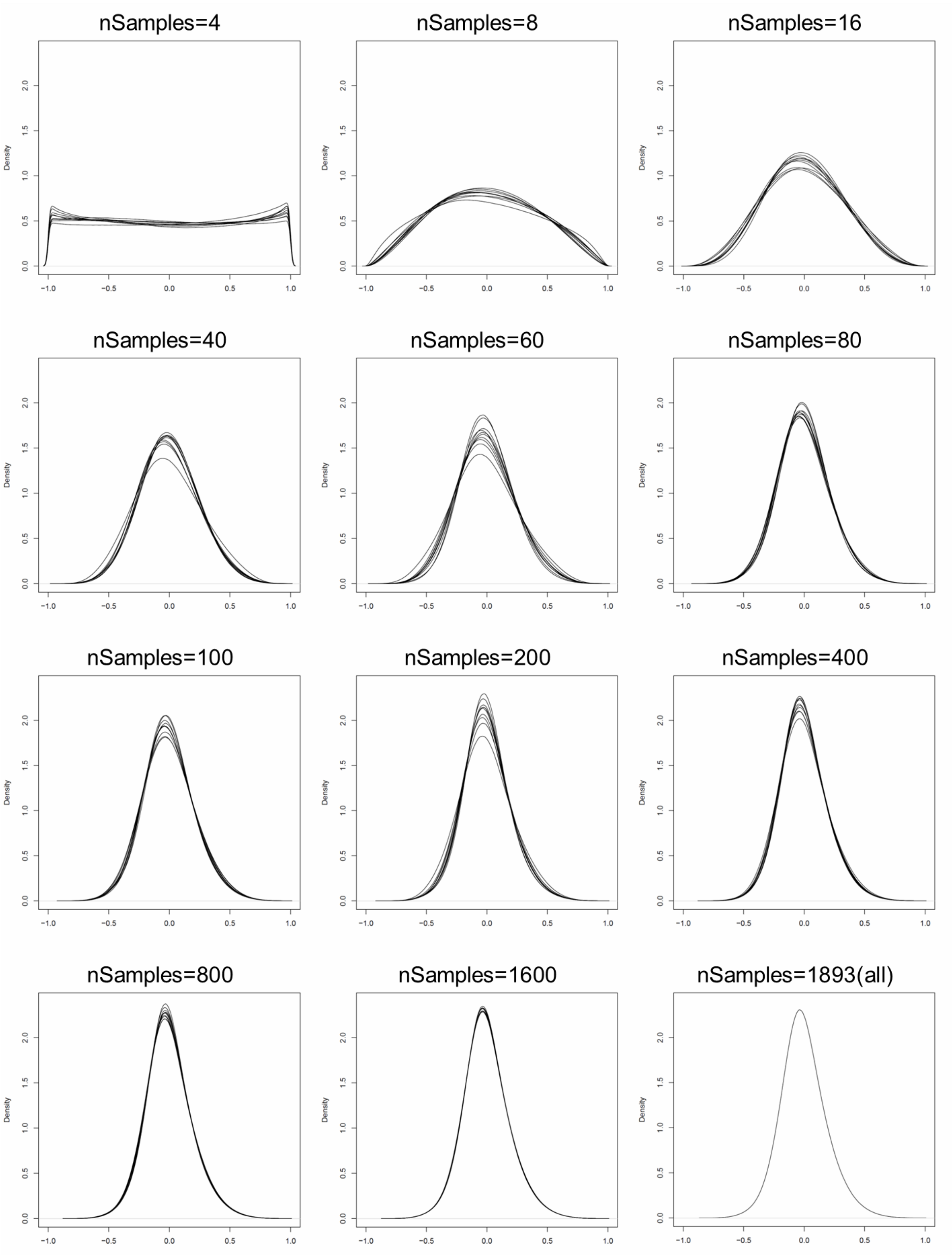

Supplemental Fig. 3. Density plots of Pearson's correlation coefficients for different sample sizes. We sampled the subsets of total 1893 samples with different sample sizes (10 times per sample size), then calculated PCCs for all pairs of TF and target genes, and then plotted them. This shows the density of PCCs conserves as the number of sample increases.
