# Supplementary material for: Transcriptional Network Analysis Reveals Drought Resistance Mechanisms of AP2/ERF Transgenic Rice
Source: Front Plant Sci. 2017 Jun 15;8:1044. doi: 10.3389/fpls.2017.01044 (PMC5471331; doi:10.3389/fpls.2017.01044)
Supplement: Supplementary file 8 [file Image4.PDF]

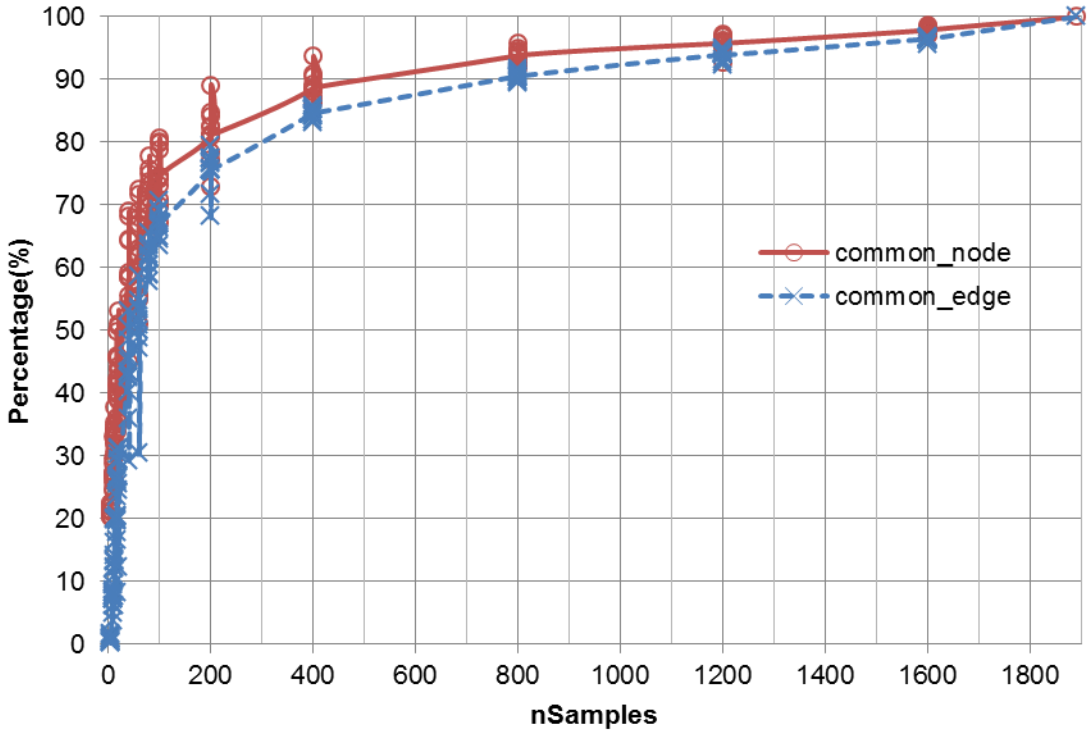


**Supplemental Fig. 4. Ratios of common nodes and edges in the networks constructed from different sample sizes.** We first tried constructing a template network from total 1893 samples by selecting edges with strong PCCs until the network contains 20% of all nodes (i.e. probes in microarray data). Then, we sampled the subsets of total 1893 samples with different sample size (10 times per a sample size), then constructed networks with the same size (the number of edges) as of the template network. Then we drew how many nodes and edges of the networks are common with the template network. This shows the network topology conserves as the number of sample increases.
