# Supplementary material for: Transcriptional Network Analysis Reveals Drought Resistance Mechanisms of AP2/ERF Transgenic Rice
Source: Front Plant Sci. 2017 Jun 15;8:1044. doi: 10.3389/fpls.2017.01044 (PMC5471331; doi:10.3389/fpls.2017.01044)
Supplement: Supplementary file 9 [file Image5.PDF]

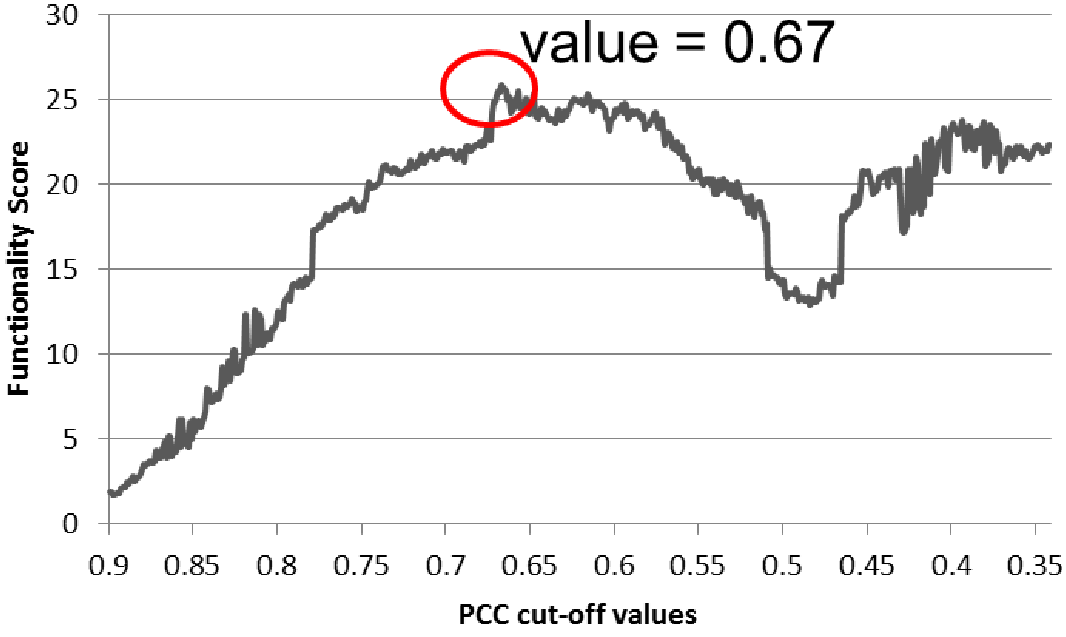

Supplemental Fig. 5. Functionality score of each network constructed using different Pearson's correlation coefficient cutoff values. This shows that the cutoff value of 0.67 maximizes functionality score. Thus, it was used for network construction in this study.
