# Supplementary material for: Transcriptional Network Analysis Reveals Drought Resistance Mechanisms of AP2/ERF Transgenic Rice
Source: Front Plant Sci. 2017 Jun 15;8:1044. doi: 10.3389/fpls.2017.01044 (PMC5471331; doi:10.3389/fpls.2017.01044)
Supplement: Supplementary file 10 [file Image6.PDF]

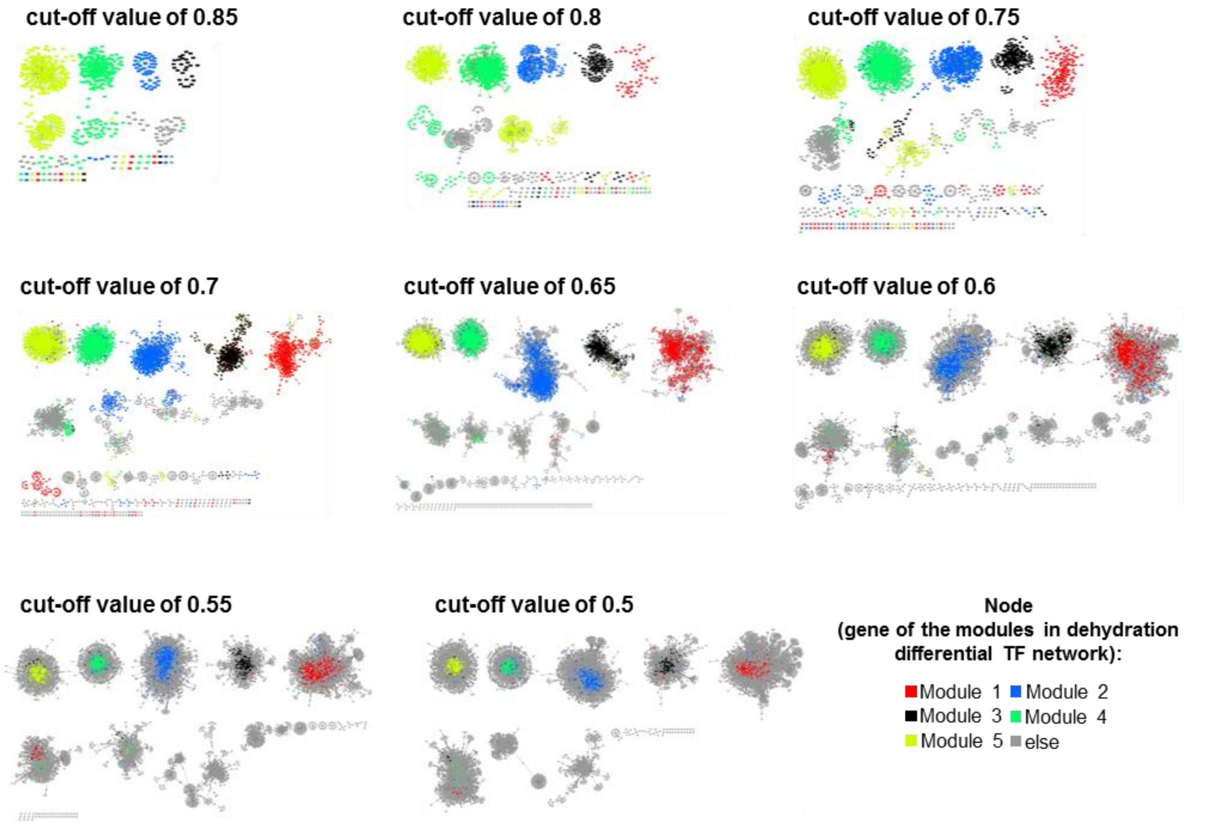

Supplemental Fig. 6. Gene clusters of transcription factor networks using different Pearson's correlation coefficient cutoff values. We calculated Pearson's correlation coefficients (PCCs) for all pairs of TF and target genes from 1893 microarrays and 8 mRNA-seqs. Varying the PCC cutoff values, we constructed networks and produced gene clusters by community clustering. The colors of nodes denote the five modules which were used in our gene module analysis. This shows gene modules are invariant (not mixed) to change of cutoff values.
