# Supplementary material for: Transcriptional Network Analysis Reveals Drought Resistance Mechanisms of AP2/ERF Transgenic Rice
Source: Front Plant Sci. 2017 Jun 15;8:1044. doi: 10.3389/fpls.2017.01044 (PMC5471331; doi:10.3389/fpls.2017.01044)
Supplement: Supplementary file 11 [file Image7.PDF]

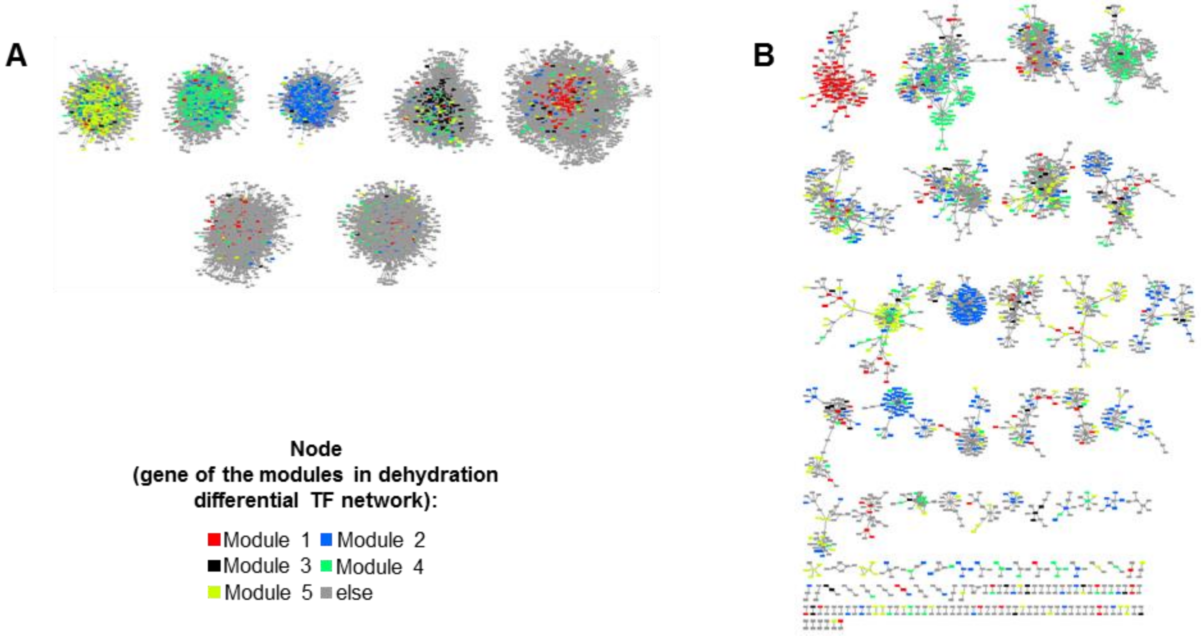

Supplemental Fig. 7. Gene clusters of Narromi- and RiceNet-based transcription factor networks. We generated dehydration TF network based on two different template networks such as (A) RiceNet and (B) Narromi. The colors of nodes denote the five modules which were used in our gene module analysis. This shows gene modules are invariant (not mixed) to change of template network for the Narromi-based network for the five modules (Module 1 to 5) but the RiceNet-based network for the one module (Module 1, red).
